# Stiff and Fracture‐Resistant Ion Gels Enabled by Synergetic Physical Entanglement and Hydrogen Bonding

**DOI:** 10.1002/smll.202509922

**Published:** 2025-10-07

**Authors:** Ryota Tamate, Yuji Kamiyama, Ken Kojio

**Affiliations:** ^1^ Research Center for Macromolecules & Biomaterials National Institute for Materials Science 1‐2‐1 Sengen Tsukuba 305‐0047 Japan; ^2^ PRESTO JST 7 Gobancho Chiyoda‐ku, Tokyo 102‐0076 Japan; ^3^ Institute for Materials Chemistry and Engineering Kyushu University Fukuoka 819‐0395 Japan; ^4^ International Institute for Carbon‐Neutral Energy Research (WPI‐I2CNER) Kyushu University Fukuoka 819‐0395 Japan

**Keywords:** entanglements, fracture resistance, hydrogen bonds, ion gels, ultrahigh molecular weight polymers

## Abstract

In this study, ion gels are developed that simultaneously exhibit exceptional stiffness and fracture resistance through the synergistic effects of physical entanglements and hydrogen bonding between polymer chains within an ionic liquid matrix. Through radical copolymerization conducted in an ionic liquid under extremely low initiator concentrations, ultrahigh molecular weight polymers in situ with nearly complete monomer conversion are successfully synthesized. This strategy enabled the one‐pot synthesis of physically crosslinked polymer gels composed of abundant entanglements and hydrogen bonds between polymer chains. Notably, it is demonstrated that the synergy between physical entanglements arising from ultrahigh molecular weight polymer chains and noncovalent hydrogen bonding enables the simultaneous enhancement of mechanical properties that typically exhibit trade‐off relationships, such as stiffness, toughness, and fracture resistance. Consequently, the synthesized ion gels exhibited outstanding mechanical performances, ranking among the best previously reported tough polymer gels, while maintaining a favorable balance between ionic conductivity and mechanical strength. These findings underscore the broader significance of the approach, indicating that the integration of physical entanglements and reversible interactions offers a generalized pathway to mechanically robust materials across various polymer systems.

## Graphical abstract

Ion gels with exceptional stiffness and toughness are fabricated by combining ultrahigh molecular weight polymer entanglements and hydrogen bonding. This synergistic strategy enables the simultaneous enhancement of typically incompatible mechanical performance, together with an unprecedented balance between mechanical robustness and ionic conductivity, thereby offering a general and versatile route to next‐generation tough and functional soft materials.

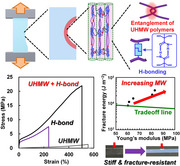

## 1 Introduction

Polymer gels are soft materials composed of a 3D polymer network swollen with a solvent. Their versatility stems from the properties of the solvent, rendering them highly promising for diverse applications, ranging from biomedical uses to electrochemical devices.^[^ ^1^, ^2^, ^3^, ^4^, ^5^, ^6^, ^7^, ^8^, ^9^ ^]^ However, a well‐known limitation of polymer gels is their inherently low mechanical strength, which arises from their high liquid content. To address this issue, various strategies have been proposed for developing tough polymer gels.^[^ ^10^, ^11^, ^12^, ^13^ ^]^ Representative examples include double‐network gels that use sacrificial bonds and tetra‐poly(ethylene glycol) hydrogels with well‐defined network structures.^[^ ^14^, ^15^, ^16^, ^17^, ^18^ ^]^ A variety of studies have also reported the enhancement of mechanical strength in ion gels using ionic liquids (ILs) as solvents, which possess unique physicochemical properties such as nonvolatility, nonflammability, and high ionic conductivity.^[^ ^19^ ^]^ For example, Hu, Dickey, and co‐workers reported that polymerizing two monomers with different solubilities in the IL led to the formation of an in situ phase‐separated structure, enabling the creation of tough and stretchable ion gels.^[^ ^20^ ^]^ Yan and co‐workers demonstrated that employing halometallate ILs, consisting of cations and coordinating anions, enabled dynamic and reversible physical crosslinking with polymer chains, thereby yielding exceptionally tough ion gels.^[^ ^21^ ^]^

In recent years, careful design of the topological structure of the polymer network has emerged as an effective strategy for enhancing the mechanical robustness of polymer gels. A pioneering example is the slide‐ring gel developed by Ito et al., which uses polyrotaxane‐based movable crosslinks.^[^ ^22^, ^23^, ^24^ ^]^ Recently, studies have developed high‐performance polymer gels by incorporating polymer chain entanglements, a ubiquitous feature of polymers, into the gel structure. Notably, the Miyata and Suo groups have independently reported the development of hydrogels with abundant entanglements by optimizing the use of chemical crosslinkers and physical entanglements within the hydrogel networks.^[^ ^25^, ^26^ ^]^

Inspired by such entanglement‐based strategies, we previously developed ultrahigh molecular weight (UHMW) ion gels, which are physical gels composed solely of entangled UHMW polymers formed in situ via radical polymerization within ILs under extremely low initiator concentrations.^[^ ^27^ ^]^ Despite the absence of chemical crosslinkers, the UHMW ion gels exhibit high mechanical stability and strength. Their network, formed exclusively through reversible chain entanglements, imparts unique properties, such as recyclability via thermal reprocessing and rapid self‐healing at room temperature via re‐entanglement of the network. Recently, several research groups have reported novel synthetic approaches for producing UHMW polymers across various polymer systems, further advancing this field.^[^ ^28^, ^29^, ^30^, ^31^, ^32^ ^]^

Despite the outstanding recyclability and self‐healing capabilities of UHMW ion gels, which rely solely on polymer chain entanglements, their mechanical strength is relatively low compared to state‐of‐the‐art tough polymer gels. In contrast, commercially adopted UHMW‐based polymers, such as UHMW polyethylene and natural rubber, exhibit remarkably high mechanical properties. UHMW polyethylene features a structure in which folded crystalline domains of polyethylene chains are interconnected by amorphous regions.^[^ ^33^, ^34^, ^35^ ^]^ For natural rubber, although its toughening mechanisms are not fully understood, strain‐induced crystallization (SIC) plays a key role in its exceptional durability.^[^ ^36^, ^37^, ^38^ ^]^

**Figure 1:**
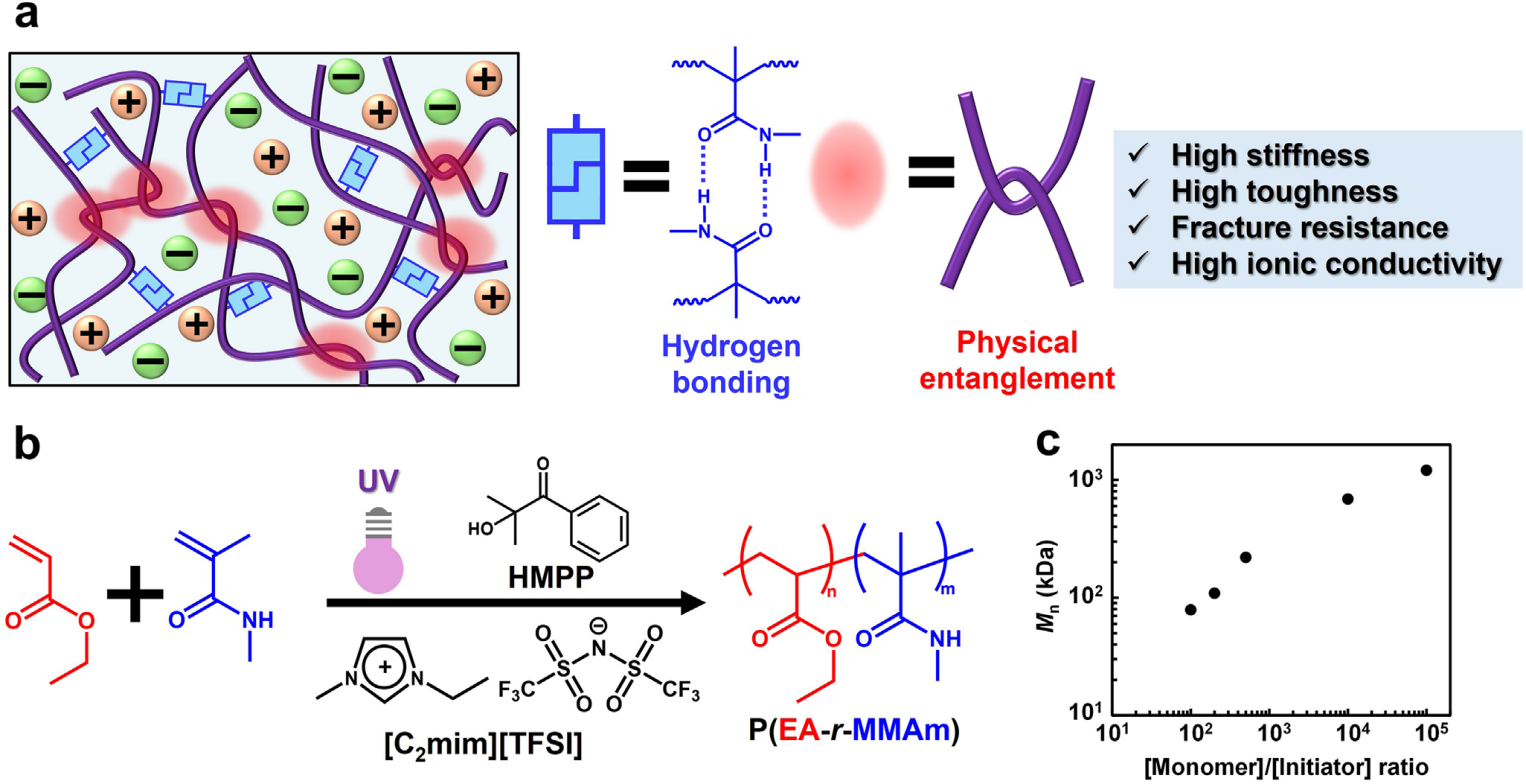
a) Schematic of an ion gel composed of hydrogen bonding and polymer chain entanglements. b) Polymerization scheme for ion gel. c) Relationship between monomer‐to‐initiator molar ratio and number‐averaged molecular weight (*M*_n_) of in situ formed P(EA‐*r*‐MMAm) copolymers in [C_2_mim][TFSI].

A common feature of these materials is the coexistence of highly entangled UHMW polymer chains and rigid nanodomains. Based on this insight, we designed a new type of polymer gel featuring dense entanglements of UHMW polymer chains and rigid nanodomains formed by noncovalent hydrogen bonding, achieved through in situ radical copolymerization within an IL (**Figure** **1**a). During gel synthesis, with a fixed monomer/IL composition, the molecular weight of the resulting copolymer can be controlled by adjusting the amount of radical initiator. Notably, we demonstrated that combining hydrogen bonding with UHMW polymer chain entanglement enables the simultaneous enhancement of physical properties that have traditionally been considered mutually exclusive, such as stiffness and fracture resistance, or mechanical strength and ionic conductivity. The synergistic toughening strategy based on physical entanglements and noncovalent interactions described here does not depend on any specific chemical structure, offering a versatile framework for designing next‐generation tough polymer materials.

## 2 Results and Discussion

We discovered that a physical gel based on non‐covalent hydrogen bonding can be formed via radical copolymerization of ethyl acrylate (EA) and *N*‐methylmethacrylamide (MMAm) in an aprotic IL, 1‐ethyl‐3‐methylimidazolium bis(trifluoromethanesulfonyl)imide ([C_2_mim][TFSI]), initiated under UV irradiation using 2‐hydroxy‐2‐methylpropiophenone (HMPP) as a photoinitiator (Figure 1b). Based on our previous finding,^[^ ^27^, ^39^, ^40^ ^]^ in the present study, we successfully synthesized hydrogen‐bonded ion gels across a wide range of molecular weights, up to the UHMW region (>10^6^ g mol^−1^), by controlling the monomer‐to‐initiator ratio through variation of HMPP concentration during UV‐induced copolymerization of EA and MMAm in [C_2_mim][TFSI] (Figure 1c). The gel permeation chromatography (GPC) traces of polymers extracted from the synthesized ion gels are shown in Figure S1 (Supporting Information). Notably, this copolymerization system results in high monomer conversion even at extremely low initiator concentrations by simply prolonging the polymerization time, thereby enabling the one‐pot synthesis of UHMW polymers. ^1^H‐NMR analysis of the ion gels after synthesis confirms that nearly all monomers are consumed, with monomer conversion exceeding 99% even in the samples containing UHMW polymers (Figure S2, Supporting Information). Details on the effect of UV polymerization time on monomer conversion are provided in the Supporting Information (Figure S3, Supporting Information). Table S1 (Supporting Information) summarizes the GPC characterization results of the hydrogen‐bonded P(EA‐*r*‐MMAm) copolymers forming the ion gels. In addition, thermal analysis was performed using differential scanning calorimetry (DSC) (Figure S4, Supporting Information). The results revealed two peaks in the derivative heat flow, indicative of glass transition temperatures, at ≈−61 and 14 °C. Similar dual DSC derivative heat flow peaks have been reported by Lodge et al. for miscible PMMA/[C_2_mim][TFSI] blend systems.^[^ ^41^ ^]^ These peaks are considered to originate from the effective local concentration associated with polymer connectivity at the length scale relevant to glass transition dynamics, as proposed by Lodge and McLeish for miscible polymer blend systems.^[^ ^42^ ^]^ In the present ternary system consisting of a copolymer and an ionic liquid, the situation is more complex; however, these peaks are presumed to arise from effective local concentrations of polymer‐rich and ionic‐liquid‐rich domains.

**Figure 2:**
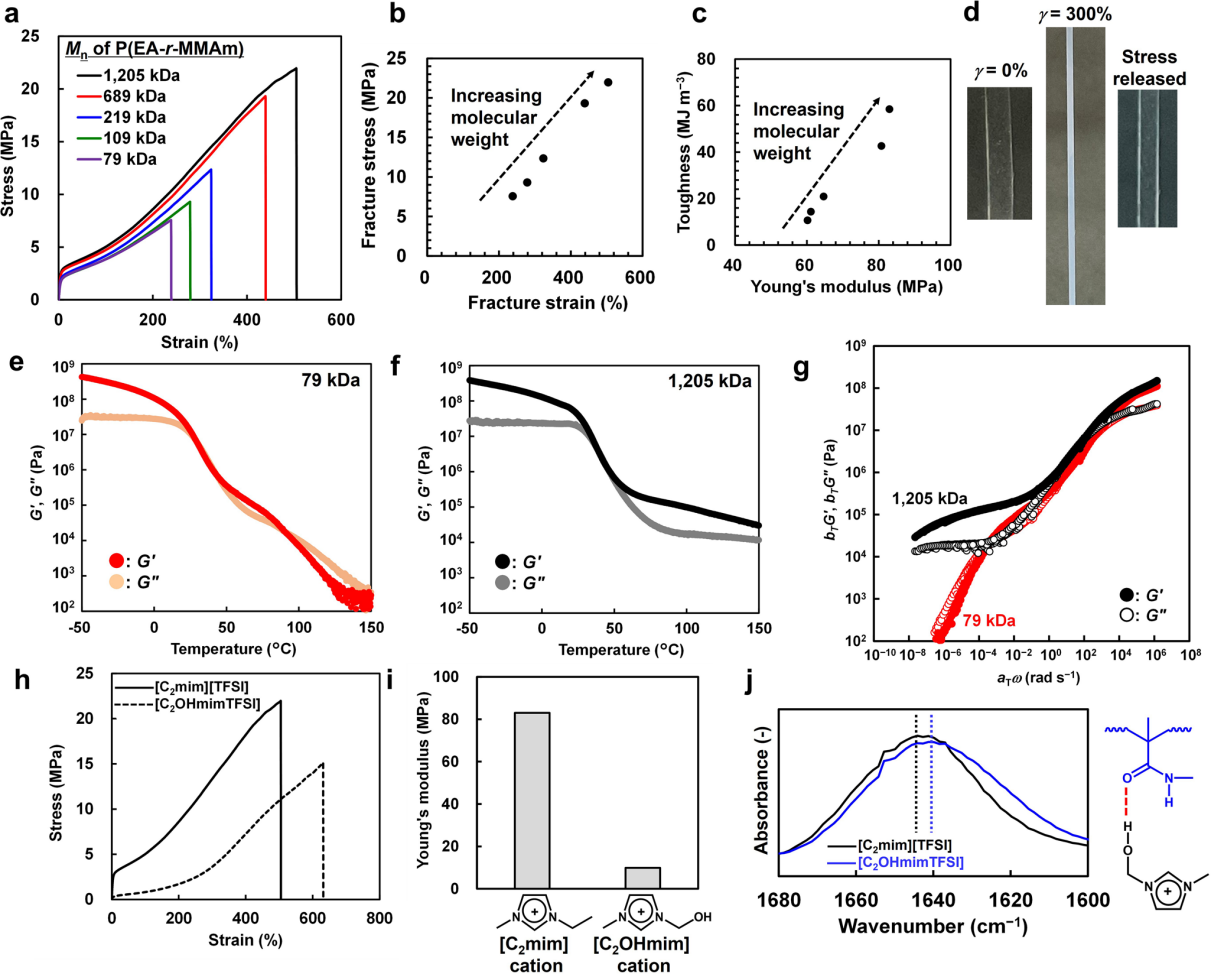
a) Uniaxial tensile tests of P(EA‐*r*‐MMAm)/[C_2_mim][TFSI] ion gels with different molecular weights. b,c) Relationship between fracture strain and stress b), and Young's modulus and toughness c) for P(EA‐*r*‐MMAm)/[C_2_mim][TFSI] ion gels. d) Strain‐induced opacification observed in P(EA‐*r*‐MMAm)/[C_2_mim][TFSI] ion gel with *M*_n_ = 1205 kDa. e,f) Temperature sweep measurements for P(EA‐*r*‐MMAm)/[C_2_mim][TFSI] ion gels with *M*_n_ = 79 and 1205 kDa, respectively. g) Viscoelastic master curves of P(EA‐*r*‐MMAm)/[C_2_mim][TFSI] ion gels obtained using the tTS principle. Reference temperature is 50 °C. h–j) Stress–strain curves h), Young's modulus i), and FTIR spectra in amide I region j) for P(EA‐*r*‐MMAm)/[C_2_mim][TFSI] and P(EA‐*r*‐MMAm)/[C_2_OHmim][TFSI] ion gels.

**Figure** **2**a shows the results of uniaxial tensile tests for P(EA‐*r*‐MMAm)/[C_2_mim][TFSI] ion gels with different molecular weights. Notably, an increase in the molecular weight of the P(EA‐*r*‐MMAm) copolymer simultaneously enhances mechanical properties that are typically considered to exhibit a trade‐off relationship, such as fracture stress–strain and Young's modulus–toughness (Figure 2b,c). In particular, the UHMW ion gel with a number‐average molecular weight (*M_n_*) of 1205 kDa exhibited exceptional mechanical performance, with Young's modulus >80 MPa, fracture stress >20 MPa, fracture strain >500%, and toughness >50 MJ m^−3^. These results suggest that the synergy between physical entanglements and non‐covalent hydrogen bonding enables simultaneous enhancement of otherwise conflicting mechanical properties. For comparison, a UHMW ion gel composed of neutral UHMW poly(methyl methacrylate) (PMMA) and [C_2_mim][TFSI], which lacks hydrogen bonding, at the same polymer fraction was considered. The gel exhibited significantly lower mechanical strength (Young's modulus of 0.5 MPa, fracture stress of 1.3 MPa, and toughness of 2.9 MJ m^−3^). Uniaxial stress‐strain curves and the resultant toughness of the ion gel composed of P(EA‐*r*‐MMAm)/[C_2_mim][TFSI] with UHMW polymers (*M*_n_ = 1205 kDa), featuring both physical entanglements and hydrogen bonding, are compared with the hydrogen‐bonded ion gel composed of P(EA‐*r*‐MMAm)/[C_2_mim][TFSI] with lower‐molecular‐weight polymers (*M*_n_ = 79 kDa), and the UHMW ion gel composed of PMMA/[C_2_mim][TFSI] (*M*_n_ = 1605 kDa) lacking hydrogen bonding (Figure S5a,b, Supporting Information). From these results, it is evident that, compared with ion gels relying on only one of these interactions, the synergistic combination of physical entanglements of UHMW polymers and hydrogen bonding enables a substantial simultaneous enhancement of mechanical properties. During tensile deformation, the initially transparent P(EA‐*r*‐MMAm)/[C_2_mim][TFSI] ion gel with *M*_n_ = 1205 kDa becomes visibly opaque upon stretching (Figure 2d). This opacification was reversible, where the gel became transparent once the stress was released. Therefore, in contrast to the crazing often observed in plastics, the reversible opacification suggests that strain‐induced structural heterogeneity, likely nanophase separation, is dynamically formed during stretching deformation.

Figure 2e,f, and Figure S6 (Supporting Information) show the temperature dependence of storage modulus (*G′*), loss modulus (*G″*) and loss tangent (tan *δ* = *G″*/*G′*) for P(EA‐*r*‐MMAm)/[C_2_mim][TFSI] ion gels with different molecular weights, *M*_n_s of 79 and 1205 kDa. In both samples, the tan *δ* peak, which is indicative of the glass transition temperature, was observed at ≈40 °C and was presumably owing to the relaxation of hydrogen bonds. At lower temperatures, no significant differences in linear viscoelasticity are observed; however, as the temperature increases, the low molecular weight sample (*M*_n_ = 79 kDa) exhibits a rapid decrease in *G′* and *G″*, and a crossover of *G′* and *G″* is observed (Figure 2e), suggesting enhanced chain mobility owing to thermal dissociation of hydrogen bonds, resulting in liquid‐like behavior. In contrast, the UHMW sample does not show a crossover of *G′* and *G″* even at 150 °C, indicating that it retains solid‐like integrities at higher temperatures (Figure 2f). This finding was likely attributed to the high entanglement density, which maintains physical crosslinks even when hydrogen bonds are thermally weakened. Figure 2g shows the viscoelastic master curves of *G′* and *G″* constructed from frequency sweep measurements at various temperatures using the time–temperature superposition (tTS) principle. Differences in low‐frequency (i.e., long‐timescale) viscoelastic behavior were clearly observed depending on the molecular weight. Although the low molecular weight sample exhibited a *G′*–*G″* crossover and terminal relaxation behavior, the UHMW sample did not exhibit any crossover within the measured frequency range, suggesting solid‐like behavior over extremely long timescales. These results demonstrated that despite being a physical gel with a characteristic relaxation time, the P(EA‐*r*‐MMAm)/[C_2_mim][TFSI] ion gel exhibited excellent shape stability. For comparison, Figures S7 and S8 (Supporting Information) show temperature sweep measurements and tTS master curves for the PMMA/[C_2_mim][TFSI] system, which lacks hydrogen bonding. Although the PMMA/[C_2_mim][TFSI] ion gel with *M*_n_ = 1626 kDa also exhibits high thermal stability and a wide plateau region owing to its abundant chain entanglements, the temperature dependence of the shift factor *a*_T_ used in tTS superposition differs significantly from that of the P(EA‐*r*‐MMAm) system (Figure S9, Supporting Information). The P(EA‐*r*‐MMAm) system exhibited considerably higher temperature sensitivity of *a*_T_ than the PMMA system. The activation energy calculated from the Arrhenius plot was *E*_a_ = 196 kJ mol^−1^ for P(EA‐*r*‐MMAm)/[C_2_mim][TFSI] compared to *E*_a_ = 106 kJ mol^−1^ for PMMA/[C_2_mim][TFSI]. Notably, molecular weight had minimal influence on *a*_T_ in either system, suggesting that the high activation energy in the P(EA‐*r*‐MMAm) system was attributed to the interpolymer hydrogen bonding.

To further investigate the role of hydrogen bonding, we performed a comparative experiment using 1‐(2‐hydroxyethyl)‐3‐methylimidazolium bis(trifluoromethanesulfonyl)imide ([C_2_OHmim][TFSI]), an IL with a hydroxyl group capable of forming hydrogen bonds, instead of [C_2_mim][TFSI]. The tensile properties of the resulting P(EA‐*r*‐MMAm)/[C_2_OHmim][TFSI] ion gel are shown in Figure 2h. Compared to that of the gel formed in [C_2_mim][TFSI], the mechanical strength was markedly reduced. Most notably, the Young's modulus decreased from ≈83 MPa to below 10 MPa (Figure 2i). FTIR spectra exhibit a shift in the amide I peak corresponding to the C═O stretching of the amide group in MMAm when the solvent is replaced with [C_2_OHmim][TFSI] (Figure 2j). This shift suggests that the hydroxyl group of [C_2_OHmim] cations formed hydrogen bonds with the carbonyl groups of MMAm, competing with the interpolymer hydrogen bonds. Similarly, a shift in the amide II peak, corresponding to C─N─H, is also observed (Figure S10, Supporting Information). These results indicate that in [C_2_OHmim][TFSI], competitive hydrogen bonding between the solvent and polymer chains disrupts interpolymer hydrogen bonding, resulting in a significant reduction in stiffness. In summary, these comparisons highlight the critical role of interpolymer hydrogen bonding in determining the mechanical performance of P(EA‐*r*‐MMAm)/[C_2_mim][TFSI] ion gels, indicating that the synergistic effects of dense physical entanglements and noncovalent bonding give rise to their outstanding mechanical properties.

**Figure 3:**
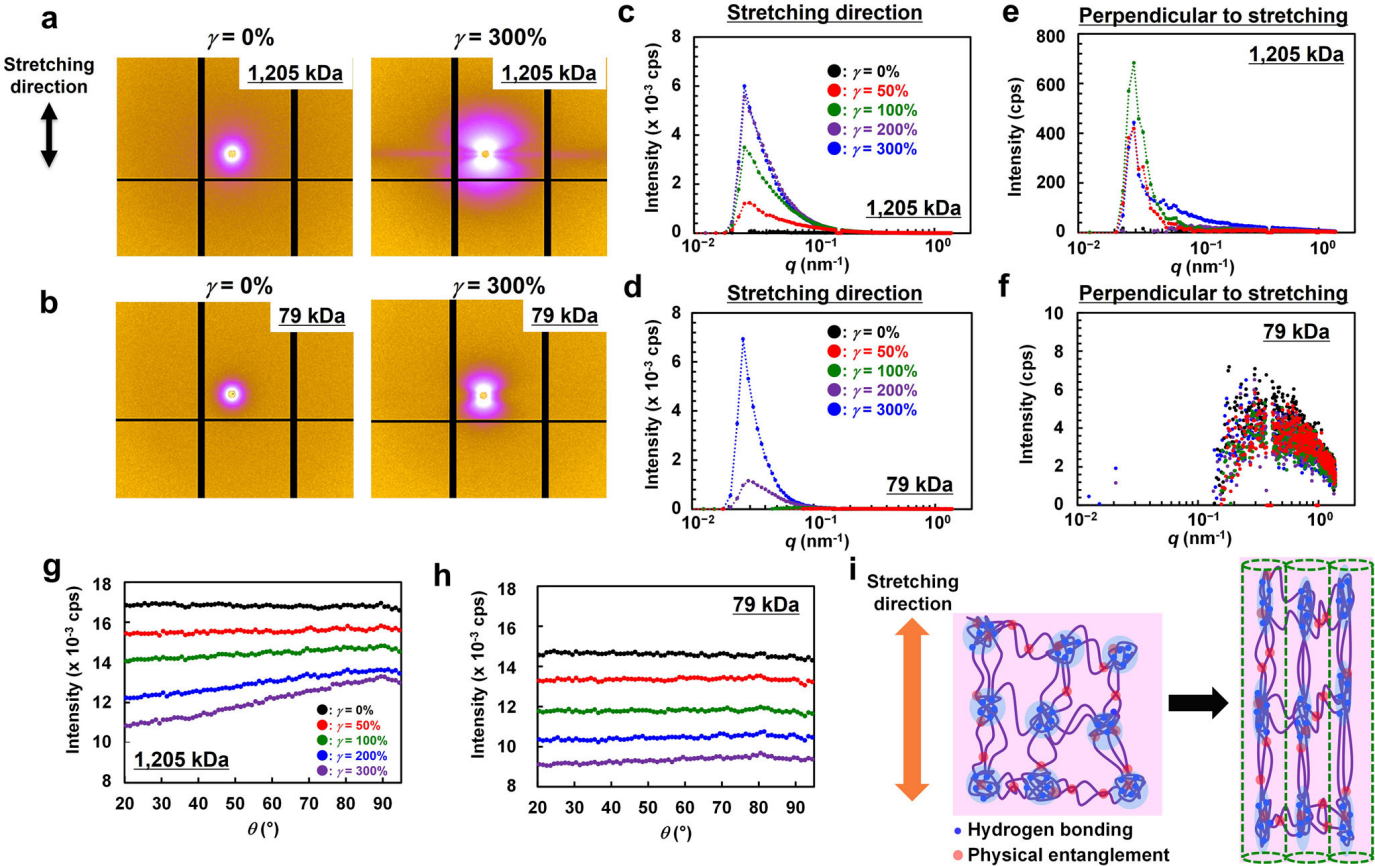
a,b) 2D SAXS patterns of P(EA‐*r*‐MMAm)/[C_2_mim][TFSI] ion gels before stretching and at 300% strain. c,d) 1D SAXS profiles along the stretching direction. e,f) 1D SAXS profiles along the perpendicular direction to stretching. a,c,e) *M*_n_ = 1205 kDa. b,d,f) *M*_n_ = 79 kDa. g,h) Azimuthal plots of 2D WAXS patterns in the *q* range of 7.8–10.5 nm^−1^. g) *M*_n_ = 1205 kDa. h) *M*_n_ = 79 kDa. i) Schematic of emergence of nanophase‐separated structures upon stretching and formation of bundled structures at high stretching ratio.

To investigate the structural evolution of ion gels during tensile deformation, we performed in situ small‐angle X‐ray scattering (SAXS) and wide‐angle X‐ray scattering (WAXS) measurements during uniaxial stretching.^[^ ^43^ ^]^ **Figures** **3**a,b, and S11 (Supporting Information) show the two‐dimensional SAXS patterns of P(EA‐*r*‐MMAm)/[C_2_mim][TFSI] ion gels with three different molecular weights upon stretching. In the unstretched state, none of the samples exhibited any discernible scattering peaks, suggesting the absence of phase‐separated structures larger than the nanometer scale typically observable in the small‐angle region. In contrast, upon stretching, scattering peaks appeared along the stretching direction as the strain increased, resulting in an “abnormal butterfly pattern.” These abnormal butterfly patterns have also been reported during the elongation of chemically crosslinked hydrogels and crystalline polymers, as well as polymer solutions under shear deformation.^[^ ^44^, ^45^, ^46^, ^47^ ^]^ This observation indicates that strain promotes structural heterogeneity within the gel,^[^ ^44^ ^]^ leading to the formation of hydrogen‐bond‐rich nanodomains that are immiscible with the IL [C_2_mim][TFSI]. Notably, in the UHMW sample, the scattering peaks appear at lower strains compared to the lower molecular weight samples, as shown in Figure S11 (Supporting Information). Figure 3c,d shows the 1D SAXS profiles along the stretching direction for the 79 and 1205 kDa samples. In both ion gels, a scattering peak appears at ≈*q* = 0.25 nm^−1^ as the strain increases. The UHMW sample exhibited this peak starting from 50% strain, whereas the low‐molecular‐weight sample required up to 200% strain for the peak to emerge. This finding suggests that strain‐induced inhomogeneity is more readily promoted in the UHMW ion gel. This behavior might be attributed to the structural relaxation in low‐molecular‐weight samples owing to rapid disentanglement upon stretching. In contrast, in the UHMW sample, the dense entanglements effectively “freeze” the structure on the timescale of the tensile test. Consequently, even at low strains, nanodomain formation is evident as the UHMW polymer chains bridge these domains. Similarly, the 219 kDa sample does not exhibit any peak up to a strain of 200% (Figure S12, Supporting Information), further supporting that UHMW polymers significantly promote the development of strain‐induced nanophase separation. Notably, only in the UHMW gel did a thin streak appear perpendicular to the stretching direction under high strains, as shown in the 2D SAXS images. This streak peak is considered to be associated with the formation of bundled structures. For example, Kojio et al. reported that in polyurethane and polythiourethane elastomers composed of hard and soft domains, a streak peak was observed in the absence of chemical cross‐linkers, which was attributed to the formation of nanofibrils resulting from the rearrangement of hard domains.^[^ ^48^, ^49^ ^]^ Figure 3e shows the 1D SAXS profiles in the direction perpendicular to stretching for the 1205 kDa sample. With increasing strain, the scattering intensity increases, suggesting a higher density of scatterers. In contrast, despite the increase in scattering intensity along the stretching direction, the lower‐molecular‐weight samples (*M*_n_s = 79 and 219 kDa) exhibit almost no scattering intensity in the perpendicular direction (Figure 3f; Figure S13, Supporting Information). While the detailed origin of this streak remains unclear, we speculate that the strain‐induced nanodomains align to form bundled structures perpendicular to the stretching direction.

In the 2D WAXS measurements during stretching, no distinct spot peaks were observed. However, the UHMW sample exhibited relatively stronger scattering intensity perpendicular to the stretching direction (*q* = 90°) compared to the lower molecular weight samples (Figure S14, Supporting Information). Recently, reported high‐strength and highly resilient gels exhibiting SIC, spot peaks corresponding to polymer chain crystallization, have been observed in WAXS patterns under stretching.^[^ ^23^, ^50^, ^51^, ^52^ ^]^ In the present ion gels, the absence of such spot peaks indicated that polymer crystallization was not observed. However, azimuthal plots of the 2D WAXS images reveal peaks in the perpendicular direction at high strains, which are absent in low molecular‐weight ion gels (Figure 3g,h; Figure S15, Supporting Information). Such molecular orientation has also been reported in polymer systems with microphase‐separated polymer systems, such as thermoplastic block copolymer elastomers.^[^ ^53^ ^]^ These SAXS and WAXS results suggest that the P(EA‐*r*‐MMAm)/[C_2_mim][TFSI] ion gels with *M*_n_ = 1205 kDa formed nanophase‐separated structures even at low strain owing to strain‐induced heterogeneity, where the hydrogen‐bond‐rich nanodomains were connected by UHMW polymers. This finding is consistent with the presence of hydrogen bonding observed in the FTIR spectra (Figure 2j; Figure S8, Supporting Information). At higher strains, these domains are further stretched into bundle‐like structures. Simultaneously, owing to suppressed entanglement relaxation, weak molecular orientation of stretched UHMW polymer chains is observed at high strain (Figure 3i). Recently, polymer gels designed to actively control phase‐separated structures to achieve mechanical functionalities have attracted increasing attention.^[^ ^20^, ^54^, ^55^, ^56^, ^57^ ^]^ There are also pioneering reports on polymer gels that exhibit deformation‐induced phase separation under stretching.^[^ ^58^, ^59^ ^]^ In this study, we report that the unique strain‐induced phase separation observed in the P(EA‐*r*‐MMAm)/[C_2_mim][TFSI] ion gel with *M*_n_ = 1205 kDa might contribute to the mechanical strength. We are currently conducting further investigations into the detailed structure–property relationships.

**Figure 4:**
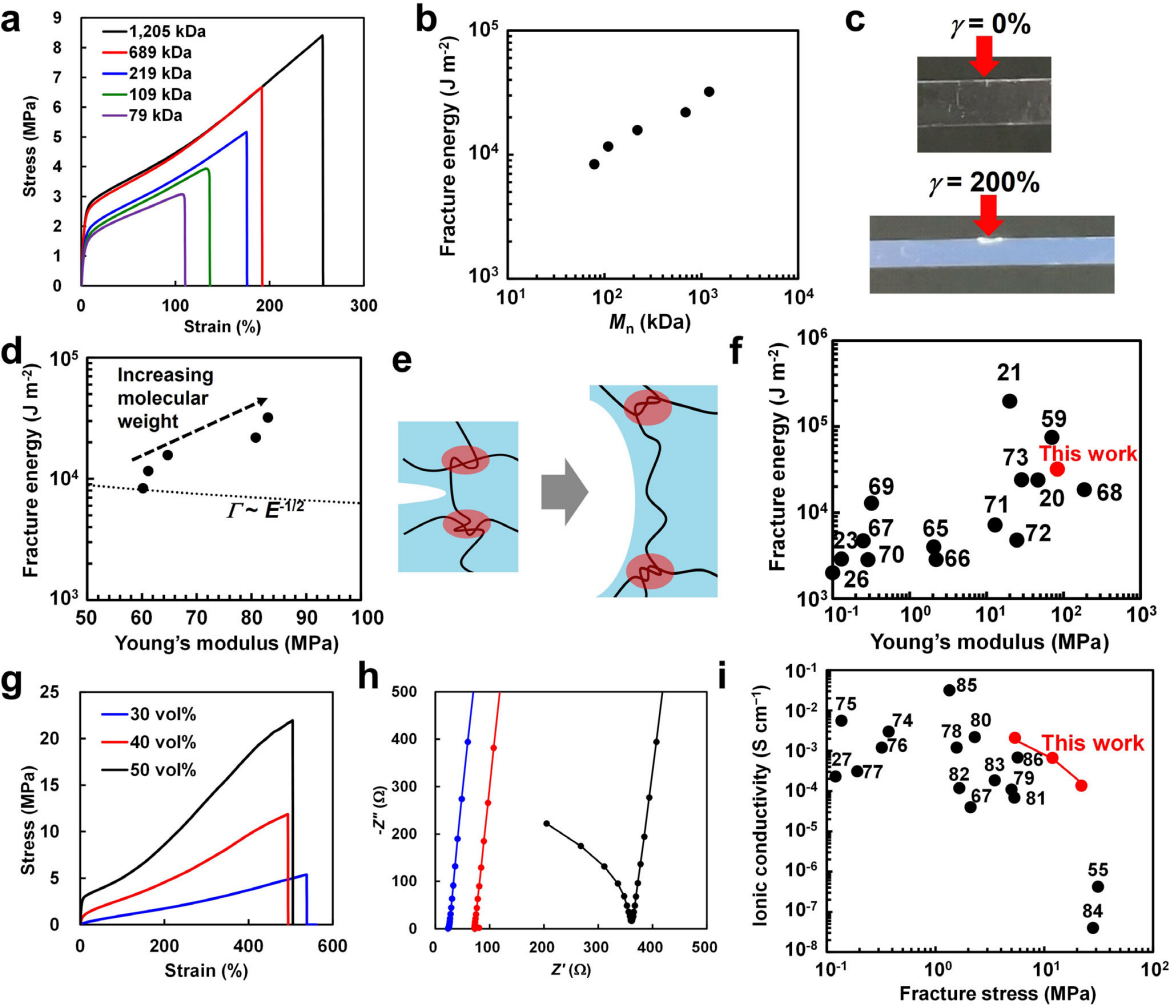
a) Uniaxial tensile tests of cracked samples of P(EA‐*r*‐MMAm)/[C_2_mim][TFSI] ion gels with different molecular weights. b) Molecular weight dependence of fracture energy for P(EA‐*r*‐MMAm)/[C_2_mim][TFSI] ion gels. c) Photographs of the tensile test of a cracked P(EA‐*r*‐MMAm)/[C_2_mim][TFSI] ion gel with *M*_n_ = 1205 kDa. d) Relationship between Young's modulus and fracture energy. Dashed line represents Lake–Thomas rule (*Γ* ∼ *E* ^−1/2^). e) Schematic of hypothesized crack tip blunting mechanism via slippage of entangled polymer chains. f) Ashby plot of Young's modulus versus fracture energy comparing previously reported tough hydrogels/ion gels (black circles) and present P(EA‐*r*‐MMAm)/[C_2_mim][TFSI] ion gel with *M*_n_ = 1205 kDa (red circle). g,h) Uniaxial tensile tests at room temperature g) and impedance measurements at 25 °C h) of P(EA‐*r*‐MMAm)/[C_2_mim][TFSI] ion gels with different polymer concentrations. i) Ashby plot of fracture stress versus ionic conductivity for P(EA‐*r*‐MMAm)/[C_2_mim][TFSI] ion gels with varying polymer concentrations (red circles) and previously reported ion gels (black circles).

To further evaluate the fracture resistance of the P(EA‐*r*‐MMAm)/[C_2_mim][TFSI] ion gels, we conducted single‐edge crack tests (**Figure** **4**a; Figure S16, Supporting Information).^[^ ^60^, ^61^ ^]^ Notably, in the P(EA‐*r*‐MMAm)/[C_2_mim][TFSI] ion gel with *M*_n_ = 1205 kDa, crack blunting is observed upon stretching the cracked samples, and the fracture energy increases with an increase in molecular weight (Figure 4b,c). Lake and Thomas formulated the fracture energy of vulcanized rubbers by considering a microscopic picture of polymers.^[^ ^62^ ^]^ They assumed that, in order to rupture polymer chains at a crack tip, the same degree of deformation must be applied to all monomer units in the vicinity of the crack. According to the Lake–Thomas model, the fracture energy *Γ* can be estimated as *Γ*∼*νLNU*, where *ν* is the number of network chains per unit volume, *L* is the displacement length at the crack tip, *N* is the degree of polymerization between cross‐linking points, and *U* is the energy required to rupture a monomer unit. If the cross‐linked polymer chains are assumed to behave as Gaussian chains, *L* scales as *N* ^1/2^, while *N* scales as *ν* ^−1^. Furthermore, by incorporating the fact that the Young's modulus *E* is proportional to the network density *ν*, according to rubber elasticity theory, the relationship *Γ*∼*E* ^−1/2^ is obtained. In other words, the fracture energy and Young's modulus can be regarded as being in a trade‐off relationship. Sakai and co‐workers verified the validity of the Lake–Thomas model for chemically cross‐linked hydrogels through experiments using tetra‐branched poly(ethylene glycol) hydrogels with uniform networks.^[^ ^63^ ^]^ In contrast, Mayumi, Ito, and colleagues demonstrated that slide‐ring gels with movable crosslinking points along the polymer chains can enhance Young's modulus without sacrificing fracture energy by increasing the crosslink density.^[^ ^64^ ^]^ This finding was attributed to the slippage of crosslinking points at the crack tip, enabling polymer chains to be pulled out from the crosslinking points. Similarly, Suo et al. have shown that hydrogels with rich entanglements can achieve high fracture energy and stiffness.^[^ ^26^ ^]^ Remarkably, in the P(EA‐*r*‐MMAm)/[C_2_mim][TFSI] ion gels, increasing the molecular weight leads to simultaneous enhancement of Young's modulus and fracture energy, overcoming the trade‐off (Figure 4d). One possible hypothesis is that the movable entanglement crosslinking points slide along the polymer backbone near the crack tip, resulting in chain pullout and crack blunting (Figure 4e). Furthermore, the nanophase separation observed under stretching in the SAXS/WAXS measurements may also occur in the highly strained region near the crack tip, potentially contributing to suppression of crack propagation (Figure S17, Supporting Information). Elucidating these mechanisms in detail remains a subject for future study. The balance between Young's modulus and fracture energy achieved here is among the highest reported for tough polymer gels, including not only ion gels but also hydrogels (Figure 4f; Table S2, Supporting Information).^[^ ^20^, ^21^, ^23^, ^26^, ^59^, ^65^, ^66^, ^67^, ^68^, ^69^, ^70^, ^71^, ^72^, ^73^ ^]^ However, the mechanical properties of the ion gels are likely to be timescale‐dependent during mechanical testing, as they rely on physical crosslinking via entanglements and hydrogen bonding, both reversible physical interactions. This consideration also applies to various tough polymer gels listed in Table S2 (Supporting Information); variations in fracture energy quantification methods and other experimental conditions also likely contribute to the differences. Therefore, Figure 4f and Table S2 (Supporting Information) should not be interpreted as definitive rankings based on absolute values.

In addition, the present gel incorporates an IL as the solvent, providing high environmental stability due to its nonvolatility and nonflammability, along with excellent ionic conductivity (Figure S18, Supporting Information). The P(EA‐*r*‐MMAm)/[C_2_mim][TFSI] ion gel with *M*_n_ = 1205 kDa exhibited a high ionic conductivity of 1.4 × 10^−4^ S cm^−1^ at 25 °C. In general, increasing polymer concentration in ion gels enhances mechanical properties but reduces ionic conductivity, indicating a trade‐off relationship. Therefore, achieving high mechanical strength and ionic conductivity remains a critical challenge in ion‐conductive polymer materials. In this system, decreasing the polymer content leads to reduced mechanical strength in tensile tests and low resistance in impedance measurements—i.e., enhanced ionic conductivity (Figure 4g,h). Nevertheless, when plotting the ionic conductivity and fracture stress of ion gels with different polymer concentrations together with previously reported high‐performance ion gels, the present system clearly demonstrates exceptional simultaneous performance in ionic conductivity and mechanical properties (Figure 4i; Table S3, Supporting Information).^[^ ^27^, ^55^, ^67^, ^74^, ^75^, ^76^, ^77^, ^78^, ^79^, ^80^, ^81^, ^82^, ^83^, ^84^, ^85^, ^86^ ^]^ Although measurement conditions vary and prevent direct comparison of absolute values, these results strongly indicate that this design concept enables the fabrication of polymer gels that combine high ionic conductivity and mechanical robustness at an outstanding level. Notably, when comparing this system with a composite of neutral polymer PMMA and [C_2_mim][TFSI], the PMMA/[C_2_mim][TFSI] system shows a considerably poorer balance between strength and ionic conductivity across different polymer concentrations, suggesting that nanophase‐separated structures may decouple mechanical strength and ionic conductivity. (Figure S19, Supporting Information). This finding further supports the effectiveness of the present concept in developing polymer gels that achieve high ionic conductivity and mechanical strength. As an example of potential applications of the ion gels, we also examined their use in strain sensing. A 30 vol% P(EA‐*r*‐MMAm)/[C_2_mim][TFSI] ion gel sheet was sandwiched between electrodes, and repeated loading–unloading cycles of strain were applied. The resistance change increased with increasing applied strain (Figure S20a, Supporting Information). Furthermore, under repeated application of 50% strain, changes in resistance could be reproducibly recorded for over 100 cycles (Figure S20b, Supporting Information). These results demonstrate that the ion gels, which combine mechanical durability with high ionic conductivity, are promising candidates for strain sensors and other flexible electrochemical devices.

## 3 Conclusion

In this study, we developed ion gels that simultaneously exhibited high stiffness and fracture resistance by leveraging the synergistic effects of physical chain entanglements and hydrogen bonding in ILs. By conducting radical copolymerization under extremely low initiator concentrations in ILs, we achieved in situ polymerization of hydrogen‐bonded UHMW polymers with high monomer conversion. This approach enabled the one‐pot synthesis of ion gels containing abundant physical entanglements and hydrogen bonds within the IL. Notably, we demonstrated that the synergistic effect between physical entanglements formed by UHMW polymers and hydrogen bonding enabled the simultaneous enhancement of mechanical properties that typically exhibit trade‐off relationships, such as Young's modulus, toughness, and fracture energy. Consequently, the developed ion gels exhibited mechanical performance ranking among the highest reported for tough polymer gels.

Our findings also highlight a broadly applicable strategy for designing high‐performance polymeric materials by combining physical entanglements with noncovalent interactions. In addition, this strategy offers the advantage of reusability, as it avoids the use of chemical crosslinkers. Furthermore, the developed ion gels also achieved an outstanding balance between ionic conductivity and mechanical strength. In recent years, materials that can simultaneously exhibit mechanical strengths and ionic conductivity have gained attention for applications such as flexible/wearable electronics,^[^ ^87^, ^88^, ^89^ ^]^ and high‐energy‐density secondary batteries where electrodes undergo large deformations during electrochemical reactions.^[^ ^90^, ^91^, ^92^ ^]^ Therefore, the present ion gels are expected to be highly useful for next‐generation electrochemical device applications.

## Conflict of Interest

The authors declare no conflict of interest.

## Supporting information



Supporting Information

## Data Availability

The data that support the findings of this study are available in the supplementary material of this article.
