# Supplementary material for: Stiff and Fracture‐Resistant Ion Gels Enabled by Synergetic Physical Entanglement and Hydrogen Bonding
Source: Small. 2025 Oct 7;21(48):e09922. doi: 10.1002/smll.202509922 (PMC12674104; doi:10.1002/smll.202509922)
Supplement: Supplementary file 1 — Supporting Information [file SMLL-21-e09922-s001.pdf]

NANO · MICRO
small

Supporting Information

for *Small*, DOI 10.1002/smll.202509922

Stiff and Fracture-Resistant Ion Gels Enabled by Synergetic Physical Entanglement and Hydrogen Bonding

Ryota Tamate, Yuji Kamiyama and Ken Kojio*

Supporting Information

Stiff and Fracture-resistant Ion Gels Enabled by Synergetic Physical Entanglement and Hydrogen Bonding

Ryota Tamate,[1,2]* Yuji Kamiyama,[1] Ken Kojio[2,3,4]

[1]Research Center for Macromolecules & Biomaterials, National Institute for Materials Science, 1-2-1 Sengen, Tsukuba, 305-0047, Japan

[2]PRESTO, JST, 7 Gobancho, Chiyoda-ku, Tokyo 102-0076, Japan

[3]Institute for Materials Chemistry and Engineering, [4]International Institute for Carbon-Neutral Energy Research (WPI-I2CNER), Kyushu University, Fukuoka 819-0395, Japan

E-mail: TAMATE.Ryota@nims.go.jp

Experimental

Materials

Ethyl acrylate (EA) was purchased from Fujifilm Wako Pure Chemical Corporation (Japan), and *N*-methylmethacrylamide (MMAm) and methyl methacrylate (MMA) were obtained from TCI (Japan). All monomers were purified by passing through a basic alumina column. 1-Ethyl-3-methylimidazolium bis(trifluoromethanesulfonyl)imide ([C_2mim][TFSI]) was purchased from Kishida Chemical (Japan), and 1-(2-hydroxyethyl)-3-methylimidazolium bis(trifluoromethanesulfonyl)imide ([C_2OHmim][TFSI]) was purchased from TCI and used as received. The photoinitiator 2-hydroxy-2-methylpropiophenone (HMPP) was also purchased from TCI and used without further purification.

Synthesis

The ion gels used in this study were synthesized via UV polymerization using a photoinitiator. The synthesis of a P(EA-*r*-MMAm)/[C_2mim][TFSI] ion gel (M_n = 1,205 kDa, monomer to initiator ([Momomer]/[Initiator]) ratio = 100,000) is described below. EA (0.81 g, 8.1 mmol), MMAm (1.35 g, 13.6 mmol), [C_2mim][TFSI] (3.45 g, 8.8 mmol), and HMPP (35.6 μg, 0.22 μmol) were weighed into a vial and stirred to obtain a

transparent precursor solution. The vial was sealed with a rubber septum and purged with argon gas for 15 min at room temperature. The precursor solution was then transferred into an argon-filled glovebox ($[H_2O]$ < 10 ppm, $[O_2]$ < 1 ppm), poured into a mold comprising a 1 mm thick silicone rubber spacer sandwiched between two PET films, and then sealed. Polymerization was carried out for 48 h inside the glovebox under 365 nm UV light using a UV lamp (intensity: ~1 mW cm^{-2}, SLUV-8, As One Corporation, Japan) to obtain the P(EA-*r*-MMAm)/[C_2mim][TFSI] ion gel.

Here, ^{1}H-NMR measurements at different polymerization times confirmed that the P(EA-*r*-MMAm)/[C_2mim][TFSI] ion gels with [Monomer]/[Initiator] ratios of 100, 200, and 500 reached monomer conversions above 99% within 0.5 h of UV polymerization, whereas the samples with ratios of 10,000 and 100,000 required more than 1 h and 6 h, respectively, to achieve conversions above 99% (Figure S3). To ensure that the vinyl peaks of the monomers were negligible in the NMR spectra of all samples, the ion gels were synthesized with polymerization times of 1 h for ratios of 100, 200, and 500, 24 h for 10,000, and 48 h for 100,000.

After polymerization, to avoid variations in properties caused by moisture absorption, the synthesized ion gels were stored in a glovebox until immediately before evaluation.

Characterization

^{1}H-NMR measurements of the ion gels were conducted using an ECS-400 spectrometer (JEOL, Japan) by directly dissolving the gels in the deuterated solvent dimethyl sulfoxide-*d*6 (DMSO-*d*6). The molecular weight of the P(EA-*r*-MMAm) copolymers was determined by gel permeation chromatography using a Prominence system (Shimadzu, Japan). To purify P(EA-*r*-MMAm) from the ion gels, the gels were first dissolved in DMSO and then reprecipitated in diethyl ether. A calibration curve was constructed using PMMA standards, and 10 mM lithium bromide in dimethylformamide was used as the eluent. FTIR spectroscopy of the ion gels was performed using an IRSpirit spectrometer (Shimadzu, Japan) equipped with an attenuated total reflectance setup with a germanium-based internal reflection element. Differential scanning calorimetry (DSC) was performed using a Discovery DSC 250 (TA Instruments, USA).

Rheological measurement

Rheological measurements were conducted using an ARES-G2 rheometer (TA Instruments, USA) equipped with an 8 mm parallel plate geometry with a cross-hatch pattern. Temperature sweep tests were performed from −50 to 150 °C at a heating rate of 1 °C min^{-1} with a constant frequency of 1 rad s^{-1}. In addition, frequency sweep

measurements were conducted every 10 °C from −50 to 150 °C over a frequency range of 0.1 to 100 rad s^{-1}. Master curves of the storage modulus (G') and loss modulus (G'') were constructed based on the time–temperature superposition principle.

Tensile tests

Uniaxial tensile tests were performed at room temperature using an AGS-X tester (Shimadzu, Japan). For noncracked samples, dumbbell-shaped specimens based on JIS K 6251 No. 7 (dimensions of the rectangular portion: 2.0 × 12.0 × 1.0 mm^3) were used, and the tests were conducted at a tensile strain rate of $\gamma = 0.14$ s^{-1}. For the single-edge crack tests, cracked samples were prepared using dumbbell-shaped specimens based on JIS K 6251 No. 8 (dimensions of the rectangular portion: 4.0 × 16.0 × 1.0 mm^3), with an initial crack of 0.6–0.7 mm introduced at the center of the sample using a razor blade. The initial crack lengths of each sample were measured by image analysis using ImageJ software (National Institutes of Health, USA).

Small-angle X-ray scattering (SAXS) and wide-angle X-ray scattering (WAXS) measurements during tensile testing

In situ SAXS and WAXS measurements during tensile testing were conducted at beamline

BL05XU of SPring-8 (JASRI, Japan). The BL05XU beamline provides a micro-focused X-ray beam with a wavelength of 0.1 nm and a beam size of 10 μm × 10 μm. During uniaxial deformation, the microbeam X-rays were irradiated onto ion gel dumbbell specimens of thickness approximately 1 mm, and corresponding 2D SAXS and WAXS patterns were acquired with an exposure time of 0.5 s each. Scattered X-rays from the sample were detected by a PILATUS 1M detector (Dectris Ltd., Switzerland) at a sample-to-detector distance of 3.90 m for SAXS and by a SOPHIAS detector (RIKEN, Japan) at a distance of 0.103 m for WAXS. 1D SAXS profiles were obtained by sector-averaging the 2D SAXS patterns along the tensile direction (azimuthal angle of 0 ± 5°) and the perpendicular direction (azimuthal angle of 90 ± 5°). For the 2D WAXS patterns, azimuthal plots were generated by radially integrating the intensity over a q range of 7.8–10.5 nm^{-1}. These profiles were obtained by integrating the 2D SAXS/WAXS patterns using the FIT2D software (Andy Hammersley/ESRF, France). The in situ SAXS and WAXS measurements were performed using a custom-built tensile tester (DIP Co., Ltd., Japan).

Ionic conductivity measurements

Ionic conductivity of ion gels was determined by impedance measurements using a VMP3

potentiostat/galvanostat (Bio-Logic Science Instruments, France). An AC voltage with amplitude 10 mV was applied over a frequency range from 0.1 Hz to 1 MHz. For the measurement cell, ion gel samples were punched into disks of diameter 16 mm and thickness of approximately 1 mm, sandwiched between stainless steel electrodes, and sealed in a two-electrode cell designed for battery evaluation (SB2A, EC Frontier, Japan). The temperature dependence of ionic conductivity was evaluated by performing impedance measurements under temperature control from 10 to 90 °C using a constant-temperature chamber.

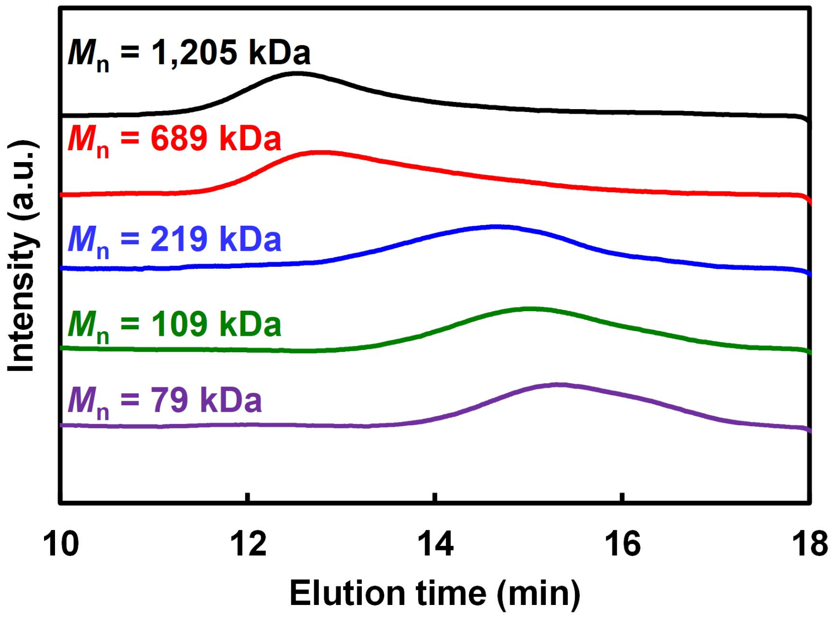

Figure S1. Gel permeation chromatography traces of P(EA-*r*-MMAm) copolymers with different molecular weights extracted from P(EA-*r*-MMAm)/[C_2mim][TFSI] ion gels.

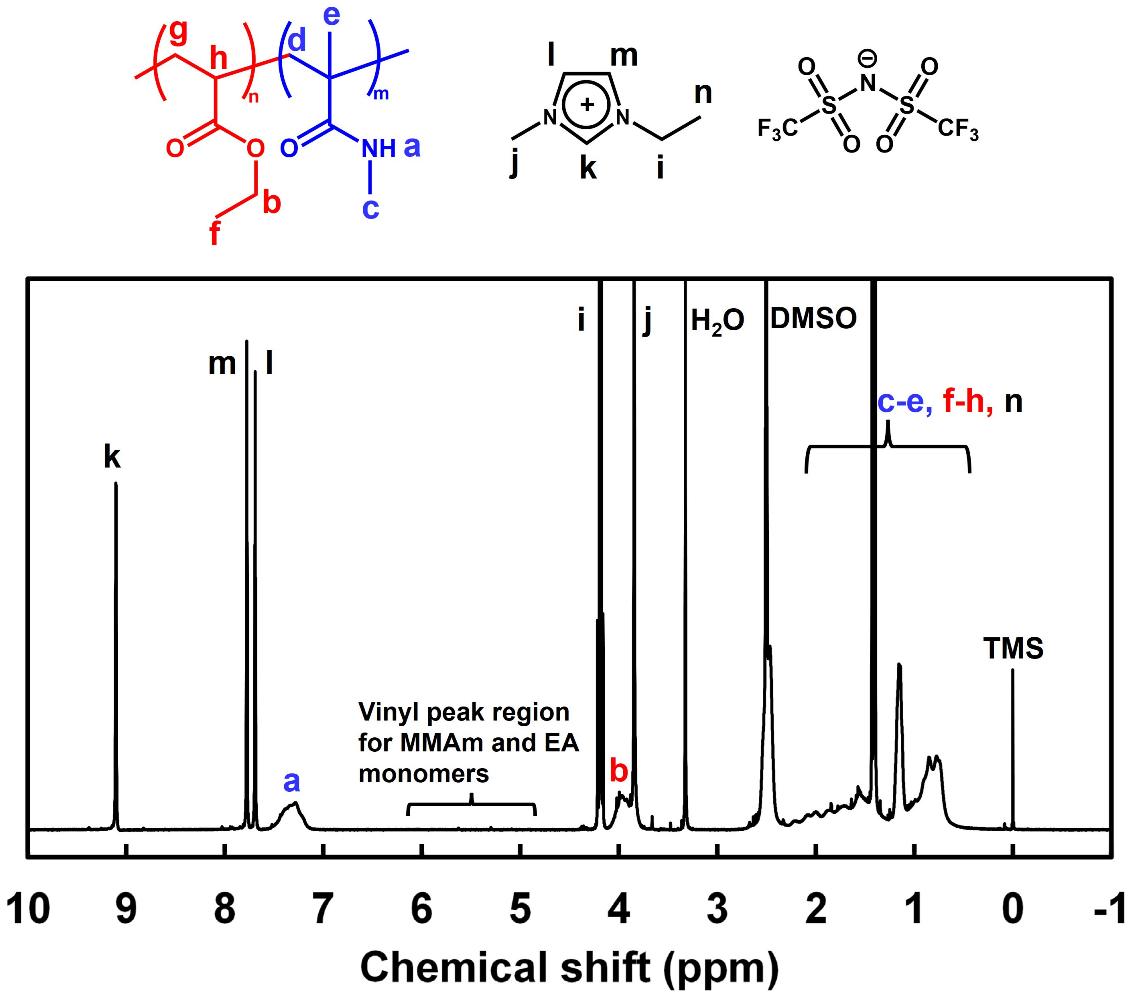

Figure S2. ^{1}H-NMR spectrum of ultrahigh molecular weight (UHMW) P(EA-*r*-MMAm)/[C2mim][TFSI] ion gel. Vinyl peaks of EA and MMAm monomers in the range 5–6 ppm are nearly absent, indicating a very high monomer conversion (> 99%) even under polymerization conditions with an extremely low initiator concentration ([Monomer]/[Initiator] = 100,000). The polymerization was carried out for 48 h.

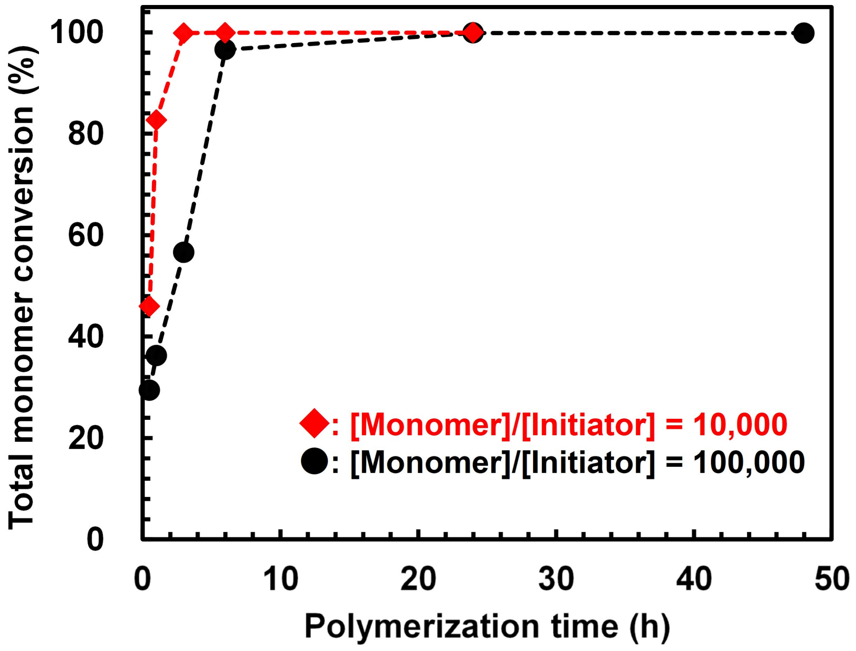

Figure S3. Relationship between polymerization time and the total monomer conversions of EA and MMAm in P(EA-*r*-MMAm)/[C_2mim][TFSI] ion gels with [Monomer]/[Initiator] ratios of 10,000 and 100,000. The total monomer conversion was calculated as the ratio of the residual [EA] + [MMAm] molar amount after polymerization, determined from the vinyl peaks of EA (dd, 1H, δ = 5.93 ppm) and MMAm (quin, 1H, δ = 5.30 ppm), to the initial total [EA] + [MMAm] molar amount before polymerization, using the C2 proton peak of [C_2mim][TFSI] (s, 1H, δ = 9.10 ppm) as an internal reference. For [Monomer]/[Initiator] ratios of 100, 200, and 500, the monomer conversion reached over 99% within 0.5 h of polymerization.

Table S1. Relationship between monomer-to-initiator ratio and molecular weight and molecular weight distribution of P(EA-*r*-MMAm) copolymers obtained by radical polymerization in [C_2mim][TFSI].

[Momomer]/[Initiator] ratio	M_n (kg/mol)	M_w (kg/mol)	Polydisiperty index
100	79	155	1.97
200	109	234	2.15
500	219	350	1.60
10,000	689	1,251	1.82
100,000	1,205	1,731	1.44

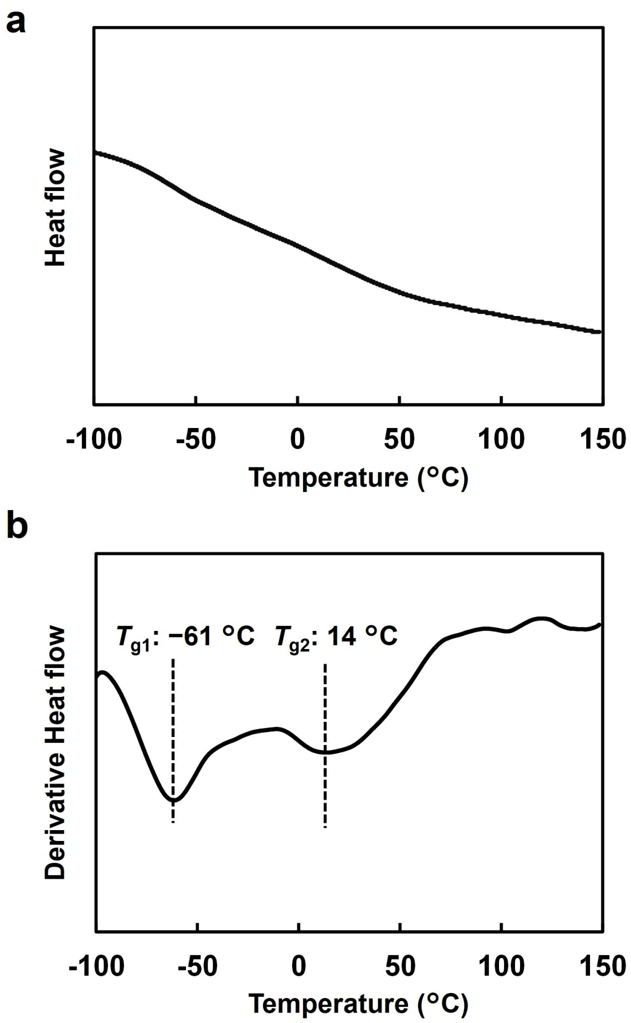

Figure S4. DSC (a) heat flow and (b) derivative heat flow curves of the P(EA-*r*-MMAm)/[C_2mim][TFSI] ion gel with M_n = 1,205 kDa.

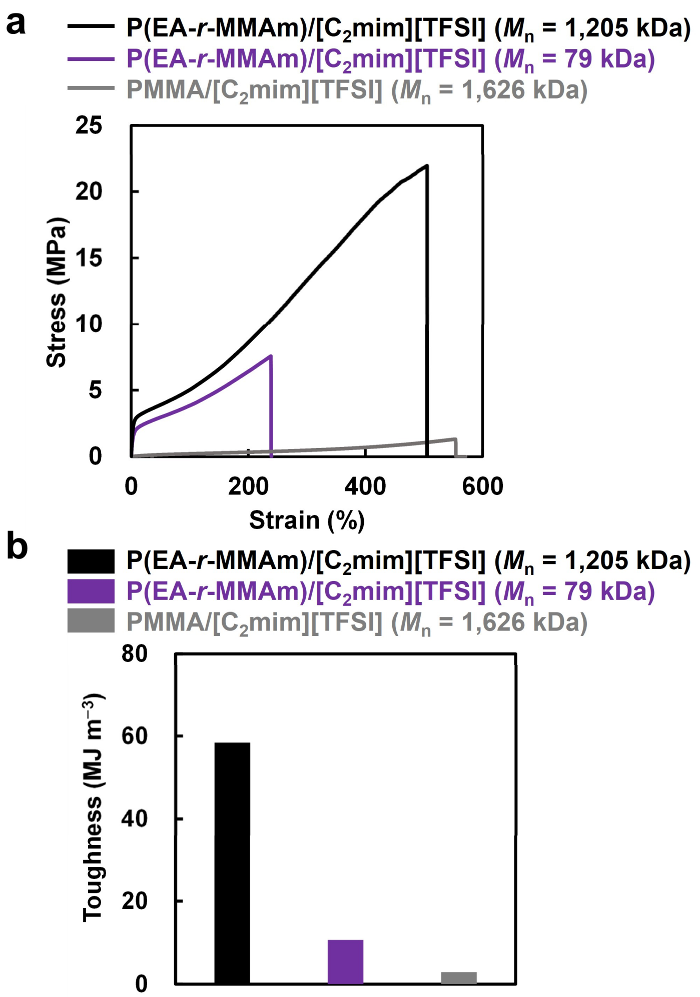

Figure S5. (a) Uniaxial tensile tests and (b) corresponding toughness of UHMW (M_n = 1,205 kDa) and low-molecular-weight (M_n = 79 kDa) P(EA-*r*-MMAm)/[C2mim][TFSI] ion gels, and UHMW (M_n = 1,626 kDa) PMMA/[C2mim][TFSI] ion gel, all at same polymer concentration ($\phi_{polymer}$ = 50 vol%).

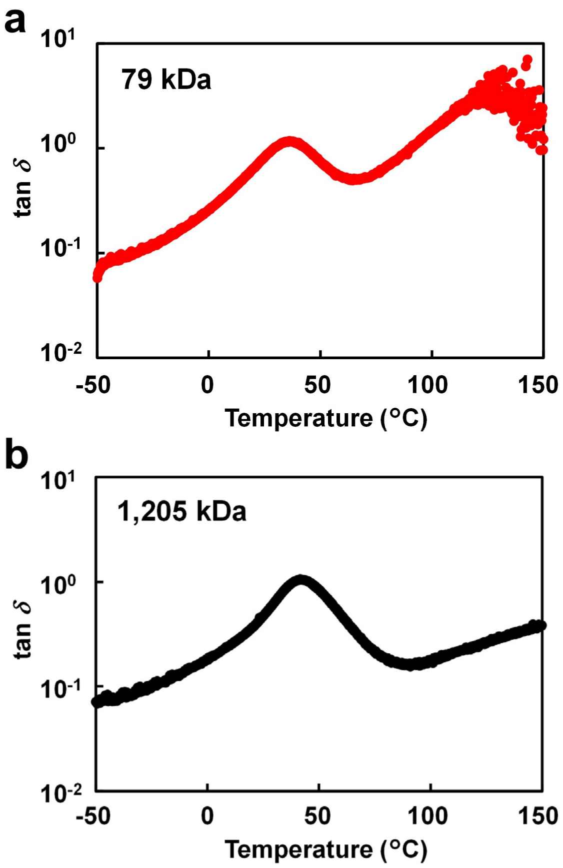

Figure S6. Temperature dependence of tan δ (= G''/G') for P(EA-*r*-MMAm)/[C2mim][TFSI] ion gels with M_n = 79 (a) and 1,205 kDa (b).

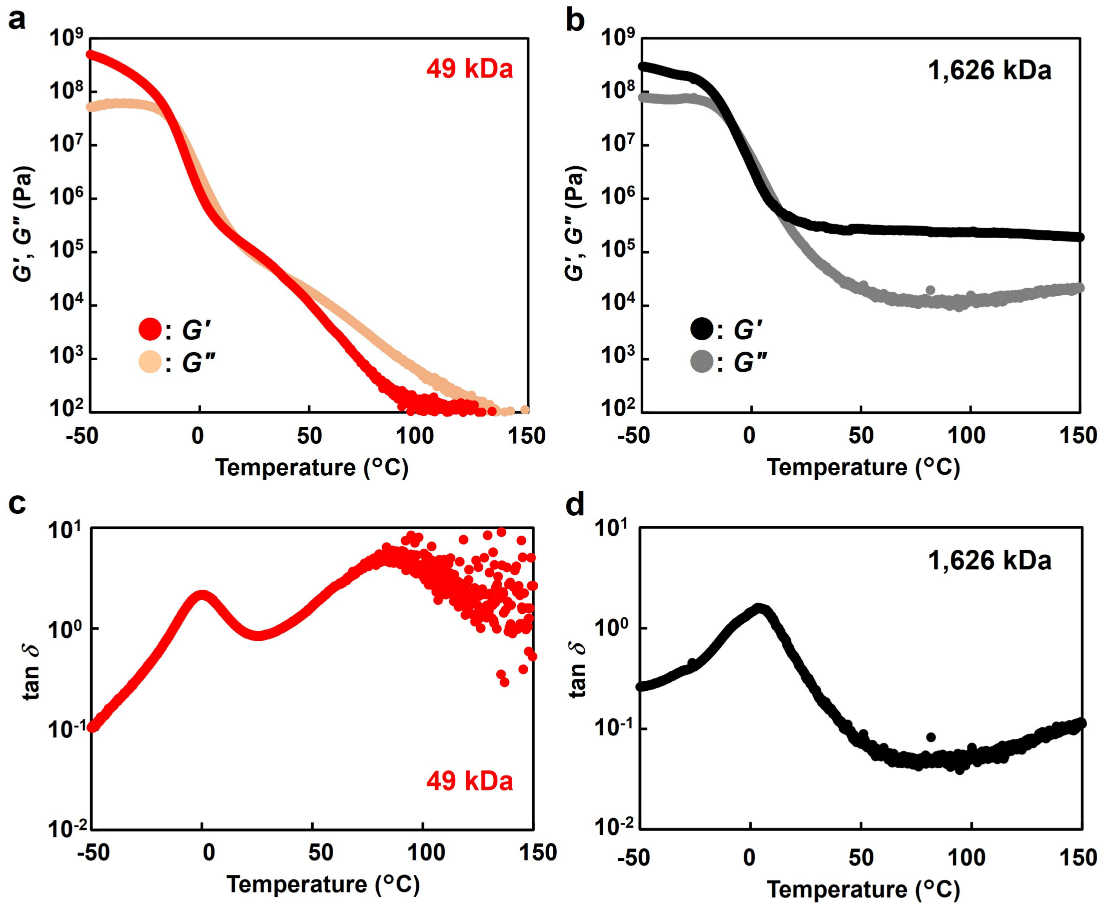

Figure S7. Temperature dependence of G' and G'' (a, b) and tan δ (c, d) for PMMA/[C_2mim][TFSI] systems with different molecular weights of M_n = 49 and 1,626 kDa.

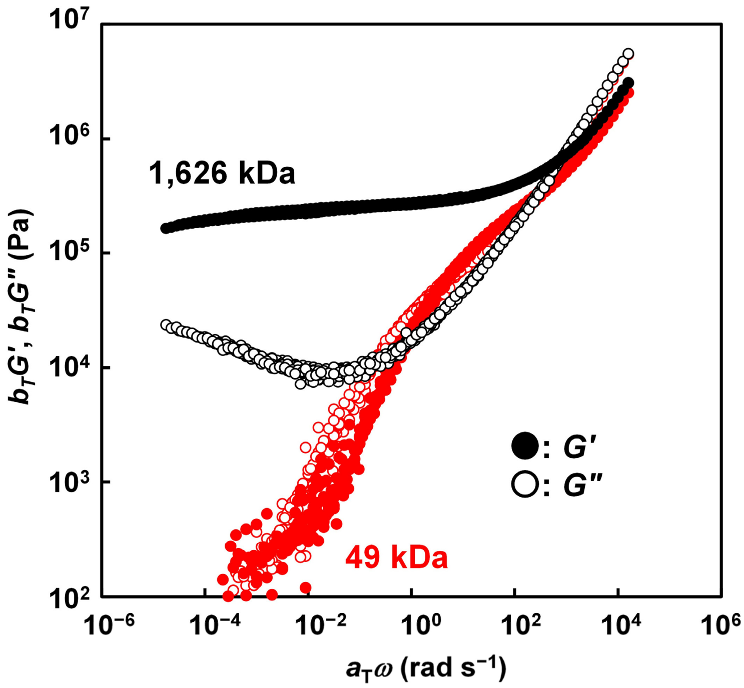

Figure S8. Viscoelastic master curves of G' and G'' obtained using time–temperature superposition principle for PMMA/[C_2mim][TFSI] systems with different molecular weights. Reference temperature is 50 °C.

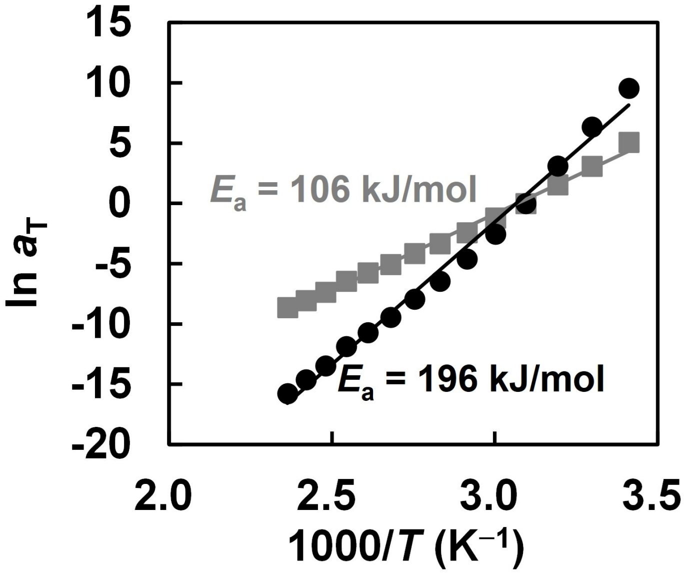

Figure S9. Arrhenius plot of shift factor a_T obtained from viscoelastic master curves for P(EA-*r*-MMAm)/[C$_2$mim][TFSI] (black circles) and PMMA/[C$_2$mim][TFSI] (grey squares), along with activation energy E_a obtained from slope of plot.

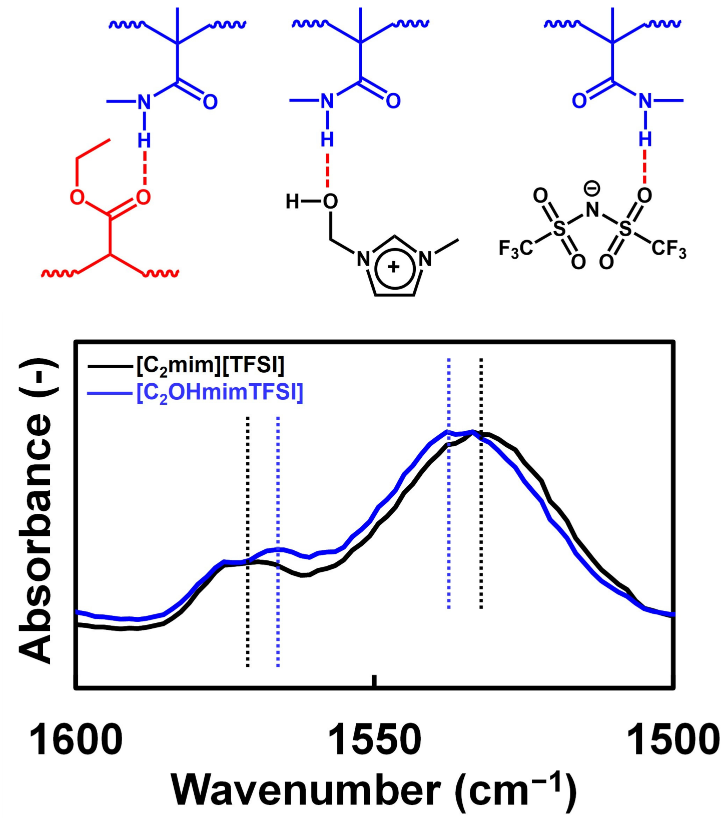

Figure S10. Comparison of amide II peak in FTIR spectra of P(EA-*r*-MMAm)/[C2mim][TFSI] ion gel (black line) and P(EA-*r*-MMAm)/[C2OHmim][TFSI] ion gel (blue line), along with proposed interactions responsible for peak shift observed in P(EA-*r*-MMAm)/[C2OHmim][TFSI] ion gel.

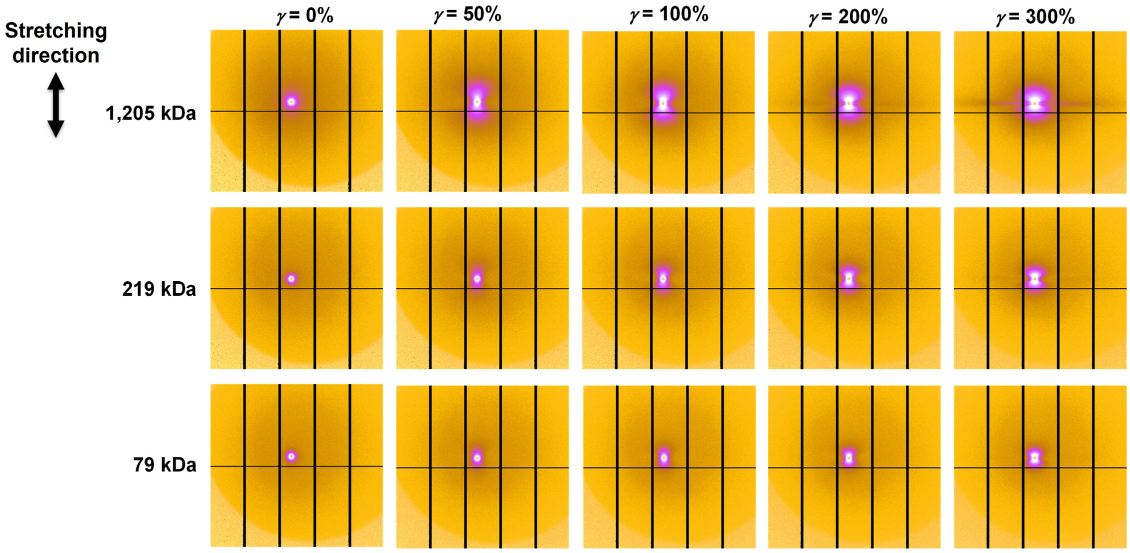

Figure S11. 2D SAXS patterns of P(EA-*r*-MMAm)/[C2mim][TFSI] ion gels with molecular weights M_n = 1,205, 219, and 79 kDa at various stretch ratios.

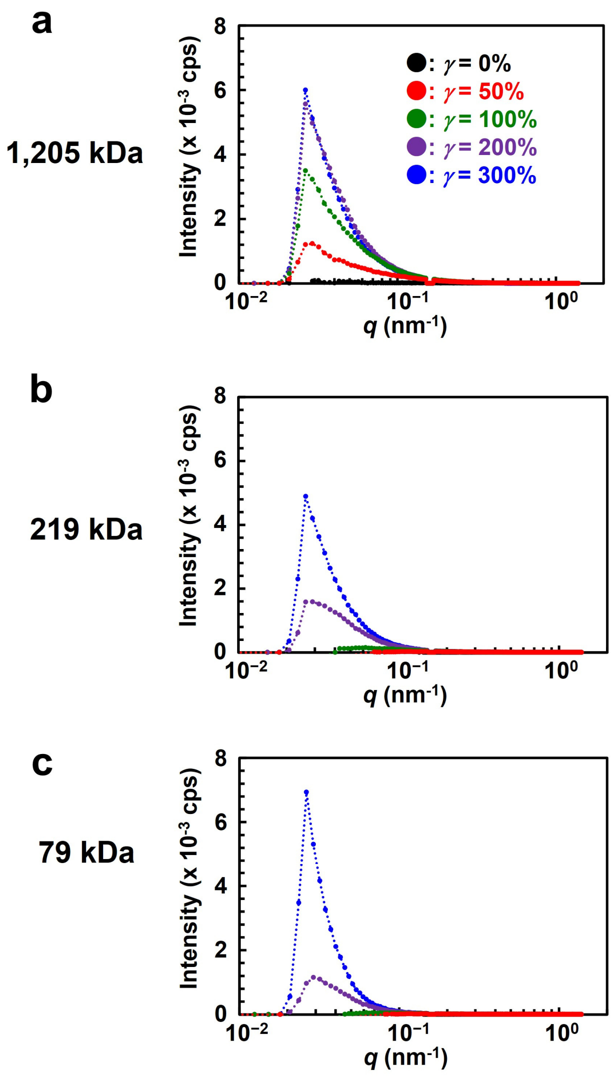

Figure S12. 1D SAXS profiles along stretching direction of P(EA-*r*-MMAm)/[C_2mim][TFSI] ion gels with (a) M_n = 1,205, (b) 219, and (c) 79 kDa.

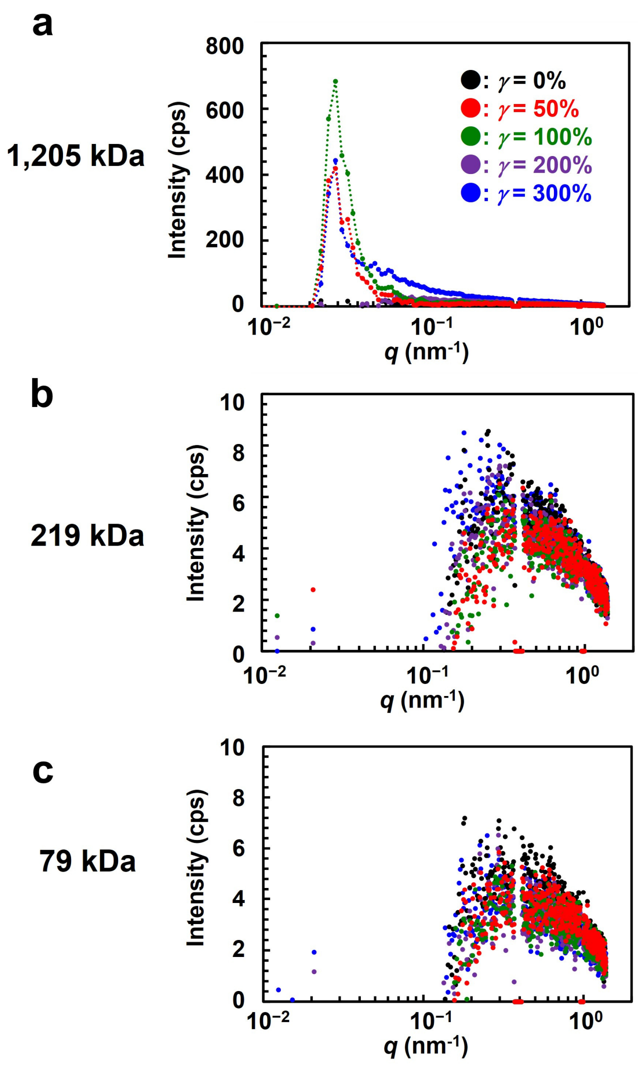

Figure S13. 1D SAXS profiles along perpendicular direction to stretching of P(EA-*r*-MMAm)/[C_2mim][TFSI] ion gels with (a) M_n = 1,205, (b) 219, and (c) 79 kDa.

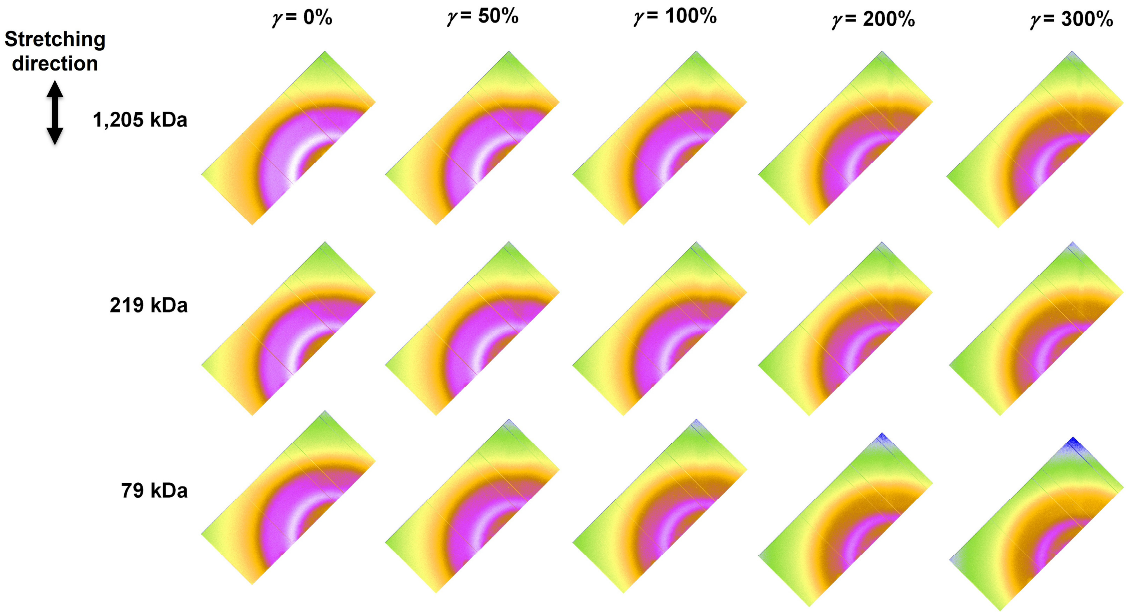

Figure S14. 2D WAXS patterns of P(EA-*r*-MMAm)/[C_2mim][TFSI] ion gels with molecular weights M_n = 1,205, 219, and 79 kDa at various stretch ratios.

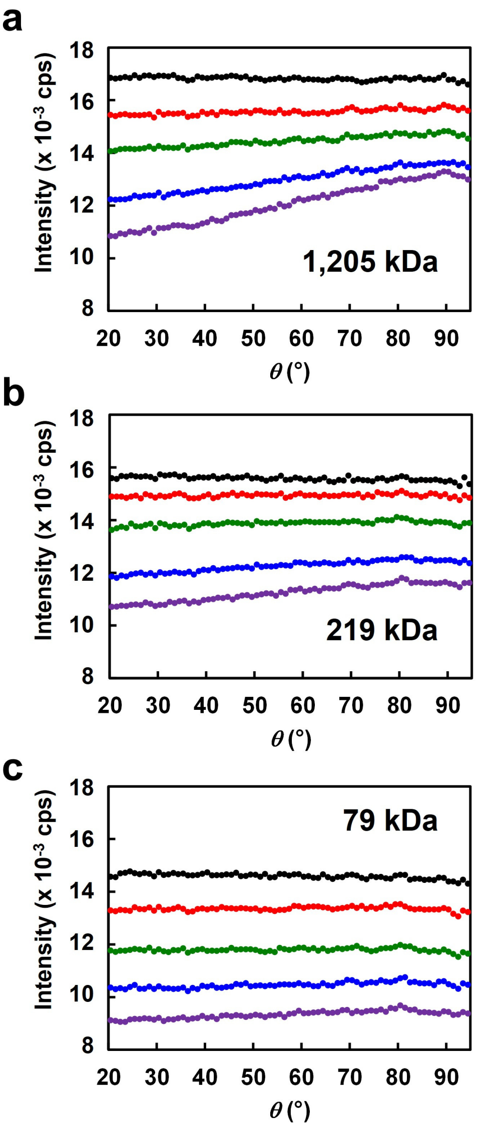

Figure S15. Azimuthal plots at q = 7.8–10.5 nm^{-1} for P(EA-*r*-MMAm)/[C_2mim][TFSI] ion gels with (a) M_n = 1,205, (b) 219, and (c) 79 kDa.

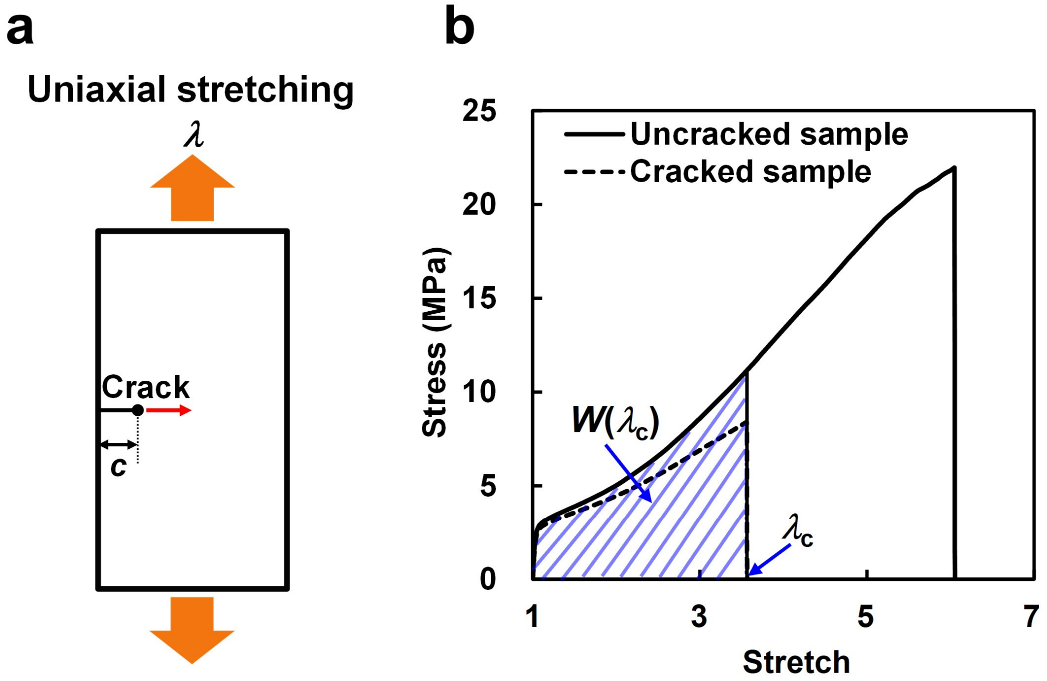

Figure S16. (a) Schematic of a cracked sample used in single-edge crack test. *c* represents initial crack length. (b) Stress–stretch curves of cracked and uncracked samples of P(EA-*r*-MMAm)/[C_2mim][TFSI] ion gels with M_n = 1,205 kDa. Fracture energy Γ is given by following equation using critical stretch ratio of cracked sample λ_c and energy density up to stretch ratio λ_c of uncracked sample (area under the stress–stretch curve) $W(\lambda_c)$:

$$\Gamma = \frac{6cW(\lambda_c)}{\sqrt{\lambda_c}}$$

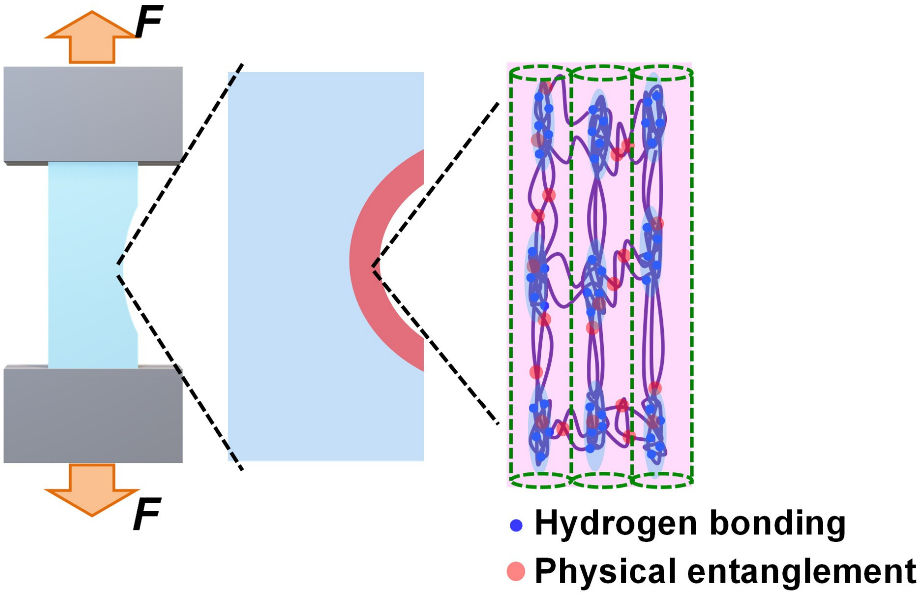

Figure S17. Schematic of hypothesis that nanophase-separated structures formed at crack tip during stretching suppress crack propagation.

Table S2. Mechanical properties of previously reported high-strength hydrogels/ion gels and those from present study.

Sample name	Young's modulus (MPa)	Fracture strain (%)	Fracture stress (MPa)	Toughness (MJ/m^3)	Fracture energy (J/m^2)	Class	Ref.
P(NaSS-*co*-MPTC)	2.1	742	1.8	7.1	4000	Hydrogel	65
B-DN3	2.2	570	11	N/A	2850	Hydrogel	66
$IG_{0.68–23\%}$	0.25	3800*	2.1*	N/A	4700	Ion gel	67
SR-0.18	0.13	1550*	1.2*	6.6	2900	Hydrogel	23
P(AAm-*co*-AA) ionogel	47	600	13	N/A	24000	Ion gel	20
IG-[Zn] (75 wt% [Bmim][ZnX])	20*	2000*	5.0*	60*	198000	Ion gel	21
MM-0.2-6	185	367	8.3	24	18500	Hydrogel	68
PAM CS-A DN gel	0.32	450*	2.1*	N/A	12900	Hydrogel	69
DN1	0.29	1000	0.92	6.4	2840	Hydrogel	70
$PNA_{1/0.1}$	71	250*	5.0*	10*	75000*	Hydrogel	59
PA hydrogel (ϕ_p = 56.6%)	13	N/A	N/A	N/A	7200	Hydrogel	71
P(AM-*co*-AA)/Na-alginate/Fe^{3+} (s-Gel)	25	220*	10	N/A	4800	Hydrogel	72
TG-25%-0.1	29	215	12	N/A	24200	Hydrogel	73
Highly entangled hydrogels (W = 2.0, C = 10^{-5})	0.10*	450*	0.39	N/A	2000*	Hydrogel	26
P(EA-*r*-MMAm)/[C_2mim][TFSI]	83	504	22	58	32092	Ion gel	This work

N/A: Not applicable. *: Values estimated from graphs in the literature.

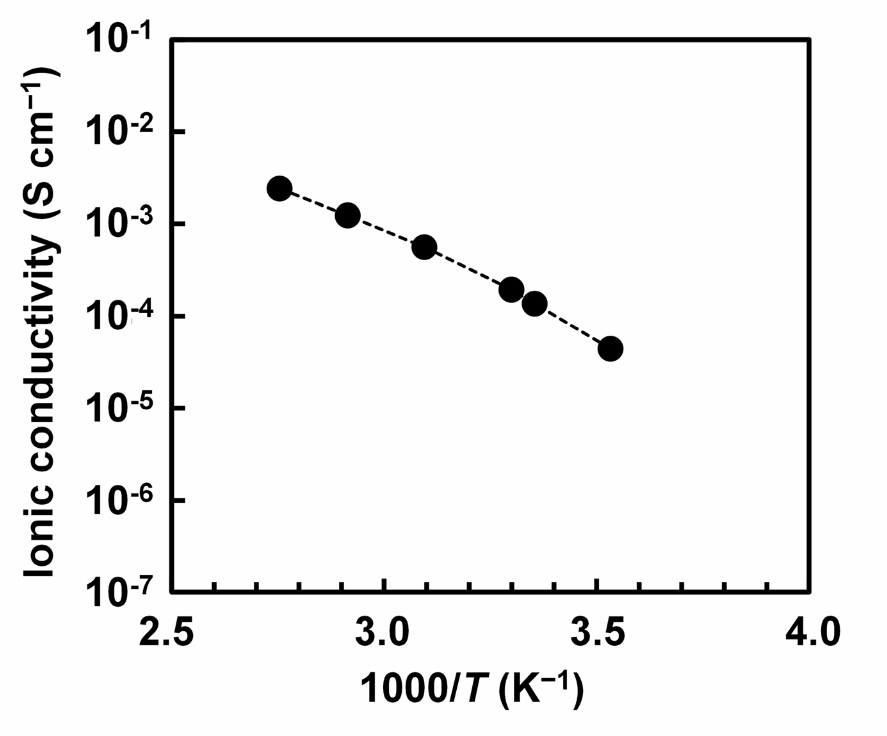

Figure S18. Temperature dependence of ionic conductivity of P(EA-*r*-MMAm)/[C_2mim][TFSI] ion gel with M_n = 1,205 kDa.

Table S3. Summary of mechanical properties and ionic conductivity of previously reported ion gels and those from present study.

Sample name	Young's modulus (MPa)	Fracture strain (%)	Fracture stress (MPa)	Toughness (MJ/m^3)	Fracture energy (J/m^2)	Ionic conductivity ($S\ cm^{-1}$)	Ref.
SOS-N_3	N/A	375*	0.40*	0.40	N/A	$3.0x10^{-3}$*	74
10 wt% tetra-PEG ion gel	0.054	737	0.14	0.59	N/A	$5.6x10^{-3}$	75
SDA ion gel	0.62	400	0.32	0.93	N/A	$1.2x10^{-3}$	76
88-PMMA-*r*-PBA	0.31	850	0.20*	N/A	N/A	$3.1x10^{-4}$	77
PU-IL2	0.42	327	1.6	N/A	N/A	$1.2x10^{-3}$	78
$IG_{0.68–23\%}$	0.25	3800*	2.1*	N/A	4700	$4.0x10^{-5}$	67
UHMW PMMA/[C_2mIm][TFSI] gel	N/A	570	0.12	0.47	N/A	$2.3x10^{-4}$	27
CNF/PVA/Borax/[EMIM][TOS]	13	300*	5.0*	N/A	N/A	$1.1x10^{-4}$	79
$IG_{70\%–10\%}$	N/A	1062	2.3	N/A	N/A	$2.2x10^{-3}$	80
MMN-35-[Li(G4)][FSI]	4.5	769	5.3	16	N/A	$6.8x10^{-5}$	81
I_{40}-SS-CPU	N/A	890	1.7	N/A	N/A	$1.2x10^{-4}$	82
$TNB_{0.75}$-50IL	1.1	495	3.5	5.5	N/A	$1.9x10^{-4}$	83
PAA-PP C_m = 6 (M)	676	388	28*	110	N/A	$4.0x10^{-8}$*	84
PVA-PAA-8M LiCl-KOH ionogel	N/A	2600	1.3	N/A	4175	$3.2x10^{-2}$	85
$HAIM_{41}$-IL_4-Ni_6	0.93	600	5.7	19	N/A	$6.9x10^{-4}$	86
PAM40-IL([EBIM]Br)	320	30	31	33	N/A	$4.2x10^{-7}$	55
30 vol% P(EA-*r*-MMAm)/[C_2mim][TFSI]	2.5	538	5.4	14	N/A	$2.1x10^{-3}$	This work
40 vol% P(EA-*r*-MMAm)/[C_2mim][TFSI]	22	493	12	29	N/A	$6.7x10^{-4}$	
50 vol% P(EA-*r*-MMAm)/[C_2mim][TFSI]	83	504	22	58	32092	$1.4x10^{-4}$	

N/A: Not applicable. *: Values estimated from graphs in the literature.

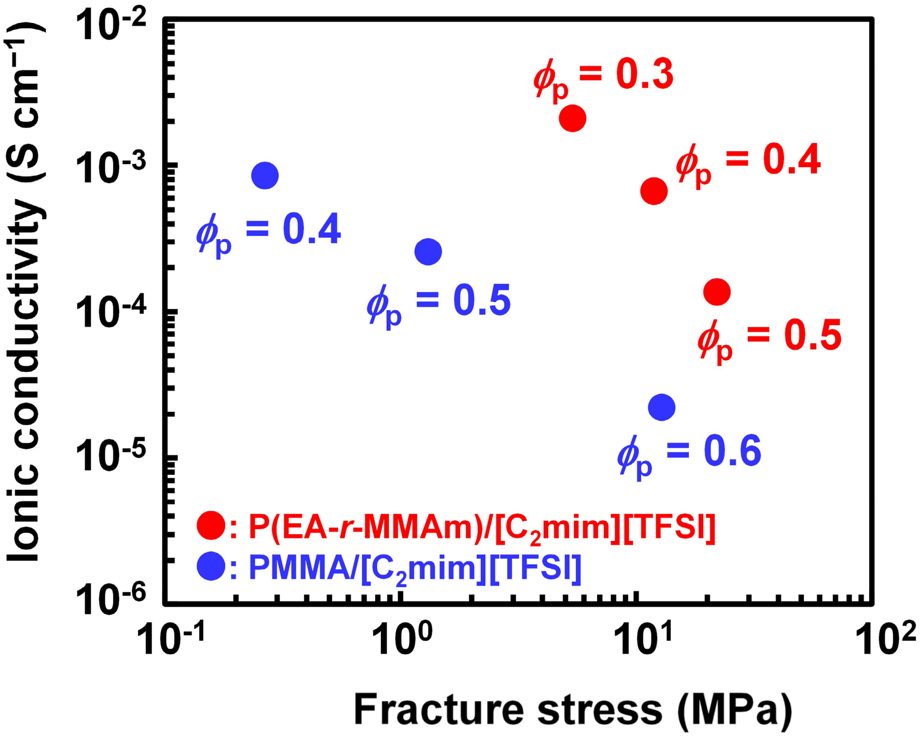

Figure S19. Relationship between fracture stress and ionic conductivity for P(EA-*r*-MMAm)/[C_2mim][TFSI] and PMMA/[C_2mim][TFSI] systems with varying polymer concentrations (ϕ_p).

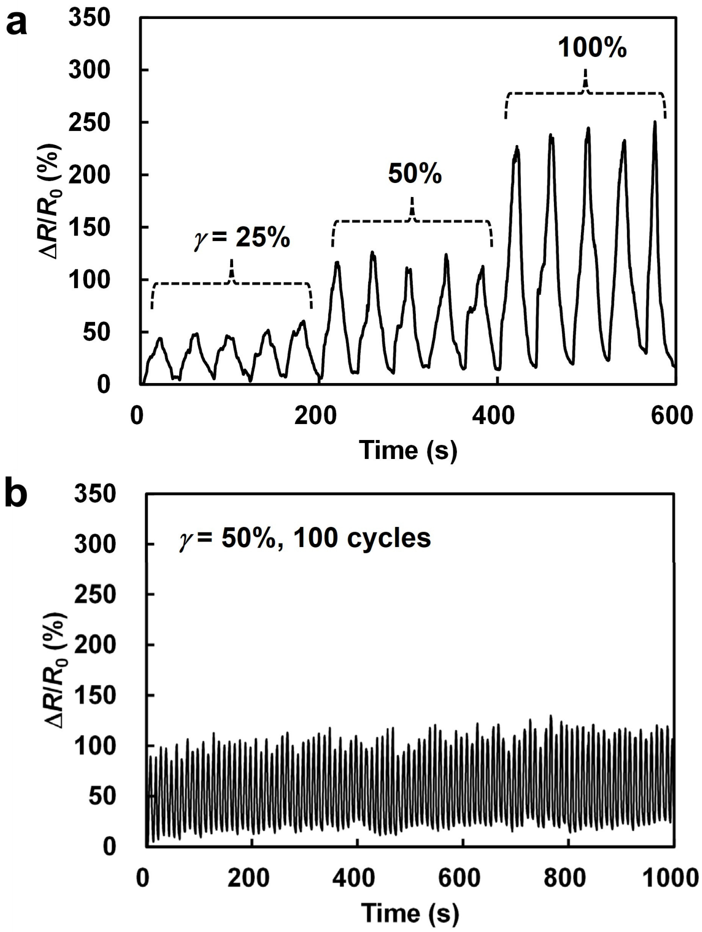

Figure S20. (a) Relative resistance changes of a 30 vol% P(EA-*r*-MMAm)/[C2mim][TFSI] ion gel sheet subjected to five manual stretching cycles at 25%, 50%, and 100% strain. (b) Relative resistance changes recorded during 100 manual stretching cycles at 50% strain.
